# Solid-solution surface alloying of Cu nanocubes with platinum-group metals: pathway switching and catalyst stabilization in CO_2_ reduction

**DOI:** 10.1039/d6sc02600a

**Published:** 2026-05-26

**Authors:** Hirokazu Kobayashi, Sachie Hikino, Akihiko Anzai, Takahiro Matsuu, Mahiru Umeno, Tomohiro G. Noguchi, Masaki Donoshita, Tomokazu Yamamoto, Yasukazu Murakami, Kenichi Kato, Takeharu Sugiyama, Hiroyuki Setoyama, Tetsuroh Shirasawa, Yuya Shimohata, Takayoshi Ishimoto, Miho Yamauchi

**Affiliations:** a Research Center for Negative Emissions Technologies, Kyushu University 744 Motooka, Nishi-ku Fukuoka 819-0395 Japan kobayashi@k-nets.kyushu-u.ac.jp; b Institute for Materials Chemistry and Engineering (IMCE), Kyushu University 744 Motooka, Nishi-ku Fukuoka 819-0395 Japan yamauchi@ms.ifoc.kyushu-u.ac.jp; c Department of Chemistry, Faculty of Science, Kyushu University 744 Motooka, Nishi-ku Fukuoka 819-0395 Japan; d The Ultramicroscopy Research Center, Kyushu University Motooka 744, Nishi-ku Fukuoka 819-0395 Japan; e Department of Applied Quantum Physics and Nuclear Engineering, Kyushu University Motooka 744, Nishi-ku Fukuoka 819-0395 Japan; f RIKEN SPring-8 Center 1-1-1 Kouto, Sayo-cho Sayo-gun Hyogo 679-5148 Japan; g Research Center for Synchrotron Light Applications, Kyushu University 6-1 Kasuga Park, Kasuga-shi Fukuoka 816-8580 Japan; h Beamline Group, SAGA Light Source 6-1 Kasuga Park, Kasuga-shi Fukuoka 816-8580 Japan; i National Institute of Advanced Industrial Science and Technology (AIST), Research Institute for Measurement and Analysis Instrumentation 1-1-1 Higashi Tsukuba Ibaraki 305-8565 Japan; j Smart Innovation Program, Graduate School of Advanced Science and Engineering, Hiroshima University 1-4-1 Kagamiyama Higashihiroshima Hiroshima 739-8527 Japan; k International Institute for Carbon-Neutral Energy Research (WPI-I2CNER), Kyushu University 744 Motooka, Nishi-ku Fukuoka 819-0395 Japan; l Advanced Institute for Materials Research (WPI-AIMR), Tohoku University 2-1-1 Katahira, Aoba-ku Sendai 980-8577 Japan

## Abstract

Precise control over morphology and alloy configuration is essential for addressing complex reactions, such as the electrochemical CO_2_ reduction reaction (CO_2_RR), which proceeds through multiple intermediates and requires enhanced selectivity, activity and stability. However, achieving simultaneous regulation of these two structural features remains a formidable challenge. Here we report novel shape-controlled Cu-based solid-solution surface-alloy nanocrystals composed of Cu nanocube (NC) cores surrounded by atomically alloyed platinum-group metal shells (Cu/Cu_1−*x*_M_*x*_ NCs, M = Pd, Pt, Ir, Ru) that alter CO_2_RR performance of Cu. In particular, surface alloying of Cu NCs with Ir switched product selectivity from C_2_H_4_ to HCOOH. Cu/Cu_1−*x*_Ir_*x*_ NCs exhibited superior HCOOH activity and stability compared with a Sn catalyst, which is a well-known element for producing HCOOH. Furthermore, Ir surface alloying preserved the cubic morphology of Cu NCs, whereas pure Cu degraded into nanograins. Our findings highlight a valuable approach to controlling reaction pathways through heteroatom interfaces and to designing highly active and stable electrocatalysts.

## Abstract 2

We report novel shape-controlled Cu-based solid-solution surface alloy nanocubes (Cu/Cu_1−*x*_M_*x*_ NCs, M = Pd, Pt, Ir, Ru) for CO_2_ reduction pathway switching and stabilization of Cu nanocube catalysts.

## Graphical abstract

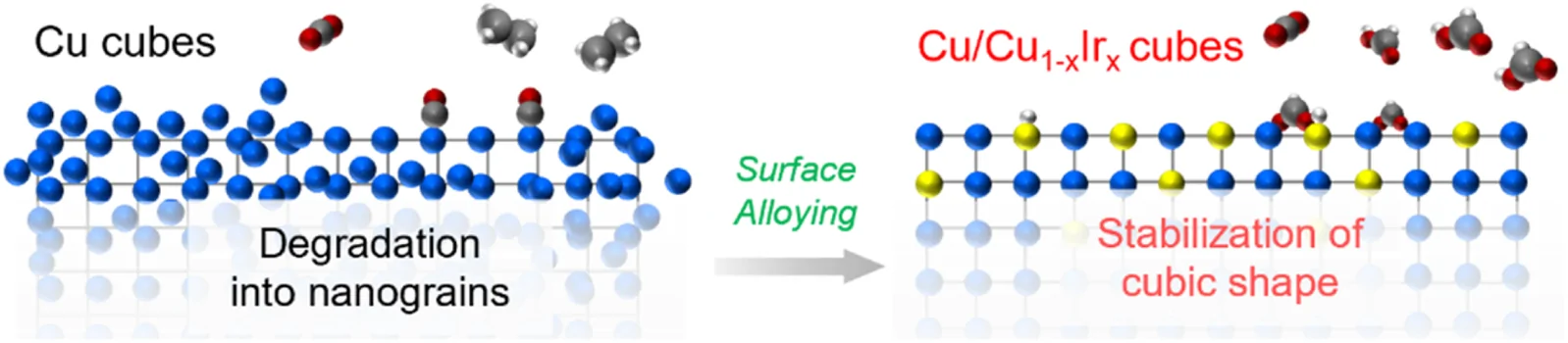

## Introduction

Nanoscale alloy catalysts exhibit distinct catalytic selectivity, activity and durability depending on their size, shape and composition.^1,2^ Solid solution alloys, in which constituent elements are homogeneously mixed at the atomic level, are particularly effective for modulating electronic states and tuning material performance through the controlled composition and combination of elements.^2,3^ In contrast, core–shell structures—typically composed of a low-cost metal core and a precious-metal shell—offer synergistic functionalities by controlling the spatial arrangement at the core–shell interface while ensuring the efficient use of scarce elements.^4,5^ Shape-controlled core–shell structures further highlight the impact of specific atomic arrangements on well-defined crystal facets.^6^ Thus, integrating the advantages of solid solution and core–shell configurations with shape control provides a promising way to achieve another dimension of catalytic functions. However, simultaneous control of morphology and surface alloy configuration in core–shell structures remains highly challenging, as both features are highly sensitive to synthetic parameters. In particular, shape-controlled solid-solution alloys composed of elements that are immiscible in the bulk state are rarely achieved. Here, we report the synthesis of a series of shape-controlled Cu-based solid-solution surface alloy nanocrystals incorporating platinum-group metals (PGMs): Cu nanocube (NC) cores surrounded by atomically alloyed PGM shells (Cu/Cu_1−*x*_M_*x*_ NCs, M = Pd, Pt, Ir, Ru) as catalysts for CO_2_ reduction. Notably, we demonstrate the formation of shape-controlled solid-solution surface alloys in Cu–Ir^7^ and Cu–Ru^8^ systems, despite their intrinsic immiscibility in the bulk state.

The electrochemical CO_2_ reduction reaction (CO_2_RR) converts CO_2_ into carbon-based chemicals,^9–11^ and Cu is unique among metals in its ability to catalyse the CO_2_RR to form a wide range of products, including methane (CH_4_), ethylene (C_2_H_4_) and ethanol (C_2_H_5_OH).^9^ However, precise control over selectivity toward specific products remains a significant challenge. Strategies to improve product selectivity on Cu have focused on faceting control,^12^ defect^13^ and strain modulation,^14^ heteroatom doping,^15^ alloying,^11,16,17^ and surface functionalization.^18^ For example, (100)-faceted Cu NCs have exhibited higher selectivity toward C_2_H_4_ compared to (111)-faceted nano-octahedra.^12^ Single-atom Cu catalysts supported on materials such as oxides have been reported to promote the formation of CH_4_.^19,20^ Bimetallic Cu–Ag systems have been shown to enhance the formation of C_2_ products, such as C_2_H_4_ and C_2_H_5_OH, compared to pure Cu.^21,22^ To date, most approaches have relied on tuning the stability of CO* and CO–CO* intermediates during the CO_2_RR.^23^ In this study, through a comprehensive investigation of CO_2_ reduction on Cu/Cu_1−*x*_M_*x*_ NCs (M = Pd, Pt, Ir, Ru), we demonstrate that solid-solution surface alloying with only 0.7 at% hydrogen evolution reaction (HER)-active Ir switches product selectivity from C_2_H_4_ to HCOOH. This alloying enables a distinct CO_2_ reduction pathway that bypasses CO* formation entirely. Remarkably, Cu/Cu_1−*x*_Ir_*x*_ NCs achieve higher activity toward HCOOH production than Sn nanoparticles (NPs), a benchmark HCOOH-selective metal catalyst, while retaining superior stability under reaction conditions. Moreover, Ir surface alloying effectively stabilizes the cubic morphology of Cu NCs, preventing the structural degradation into metallic nanograins that has been observed for pure Cu.^24,25^ Our multiple structural control strategy provides a powerful framework for tailoring nanocatalysts, addressing not only the CO_2_RR but also other electrocatalytic reactions.

## Results and discussion

We synthesized Cu/Cu_1−*x*_M_*x*_ (M = Pd, Pt, Ir, Ru), in which the shell portion of Cu NCs is atomically alloyed with M through a galvanic replacement reaction. First, uniform Cu NCs with an average edge length of 50.5 ± 5.3 nm were synthesized by a previously reported method (Fig. S1a and S2a).^12^ Subsequently, a controlled amount of an acetylacetonate complex in oleylamine, which serves as a metal precursor, was added dropwise to the solution and stirred at an appropriate synthesis temperature and for an appropriate time.

Scanning electron microscopy (SEM) and transmission electron microscopy (TEM) images revealed that the morphologies of the obtained Cu/Cu_1−*x*_Pd_*x*_, Cu/Cu_1−*x*_Pt_*x*_ and Cu/Cu_1−*x*_Ir_*x*_ NCs were cubic as was that of Cu NCs, indicating that the cubic shapes of Cu NCs were retained after the galvanic replacement reaction with Pd, Pt and Ir ions (Fig. S1b–d and S2b–d). The average edge length of Cu/Cu_1−*x*_Pd_*x*_, Cu/Cu_1−*x*_Pt_*x*_ and Cu/Cu_1−*x*_Ir_*x*_ NCs was estimated to be 50.3 ± 6.5, 56.3 ± 7.2 and 51.0 ± 8.1 nm, respectively. The average edge length of Cu/Cu_1−*x*_Ir_*x*_ NCs is in good agreement with an edge length distribution of 49.8 ± 7.6 nm estimated from small-angle X-ray scattering (SAXS) (Fig. S3). From SEM–energy-dispersive X-ray (EDX) analyses, the atomic percentages of Pd, Pt and Ir included in Cu/Cu_1−*x*_Pd_*x*_, Cu/Cu_1−*x*_Pt_*x*_ and Cu/Cu_1−*x*_Ir_*x*_ NCs were calculated to be 1.1, 1.0 and 0.7%, respectively.

**Fig. 1:**
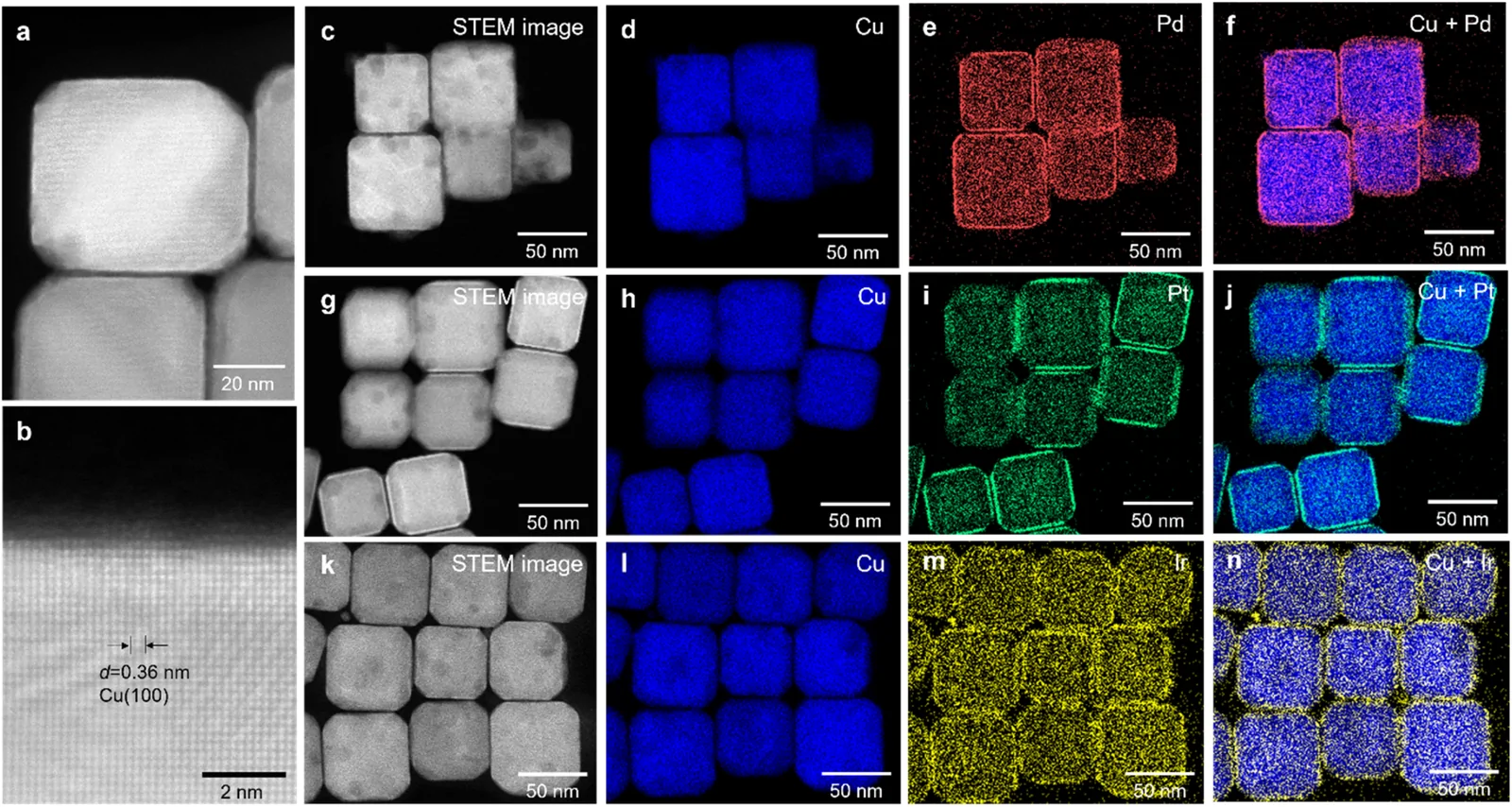
(a and b) High-resolution HAADF-STEM images of Cu/Cu_1−*x*_Ir_*x*_ NCs. (c–f) HAADF-STEM images and EDX maps of Cu/Cu_1−*x*_Pd_*x*_, (g–j) Cu/Cu_1−*x*_Pt_*x*_ and (k–n) Cu/Cu_1−*x*_Ir_*x*_ NCs. In the EDX maps, Cu, Pd, Pt and Ir are presented in blue, red, green and yellow, respectively.

To investigate the alloying states in the obtained NCs, high-resolution annular high-angle dark-field scanning TEM (HR-HAADF-STEM) and EDX elemental mapping of Cu and M were performed. HR-HAADF-STEM images of Cu/Cu_1−*x*_M_*x*_ NCs (M = Pd, Pt, Ir) clearly display atomic planes with a *d*-spacing of approximately 0.36 nm in the cubic core region, consistent with the (100) planes of the face-centered cubic (fcc) Cu structure (Fig. S4, S5 and 1a, b). The brighter atomic columns observed in the shell region, due to the higher atomic number of M compared to Cu, suggest that M atoms are atomically dispersed in the surface region of the Cu matrix (Fig. S4, S5 and 1a, b). The thickness of the Cu_1−*x*_M_*x*_ shell ranges from 4 to 8 atomic layers (1–2 nm) (Fig. S4, S5 and 1a, b). The STEM–EDX elemental mapping of Cu and the corresponding M elements (M = Pd, Pt, Ir) for Cu/Cu_1−*x*_M_*x*_ NCs, shown in Fig. 1c–n, demonstrated that Pd, Pt and Ir atoms are homogeneously distributed near the surface of the Cu NCs, while Cu atoms are distributed across the entire NCs, forming a solid-solution surface alloy. The atomic percentages of Pd, Pt and Ir contained in Cu/Cu_1−*x*_Pd_*x*_, Cu/Cu_1−*x*_Pt_*x*_ and Cu/Cu_1−*x*_Ir_*x*_ NCs were calculated to be 1.9, 1.3 and 1.0%, respectively, which are in agreement with the atomic percentages estimated from the SEM–EDX data. The STEM–EDX mapping data of the selected Cu_1−*x*_M_*x*_ shell regions revealed that the alloy compositions were estimated to be Cu_0.91_Pd_0.09_, Cu_0.92_Pt_0.08_, and Cu_0.94_Ir_0.06_, respectively (Fig. S6–S8 and Tables S1–S3).

**Fig. 2:**
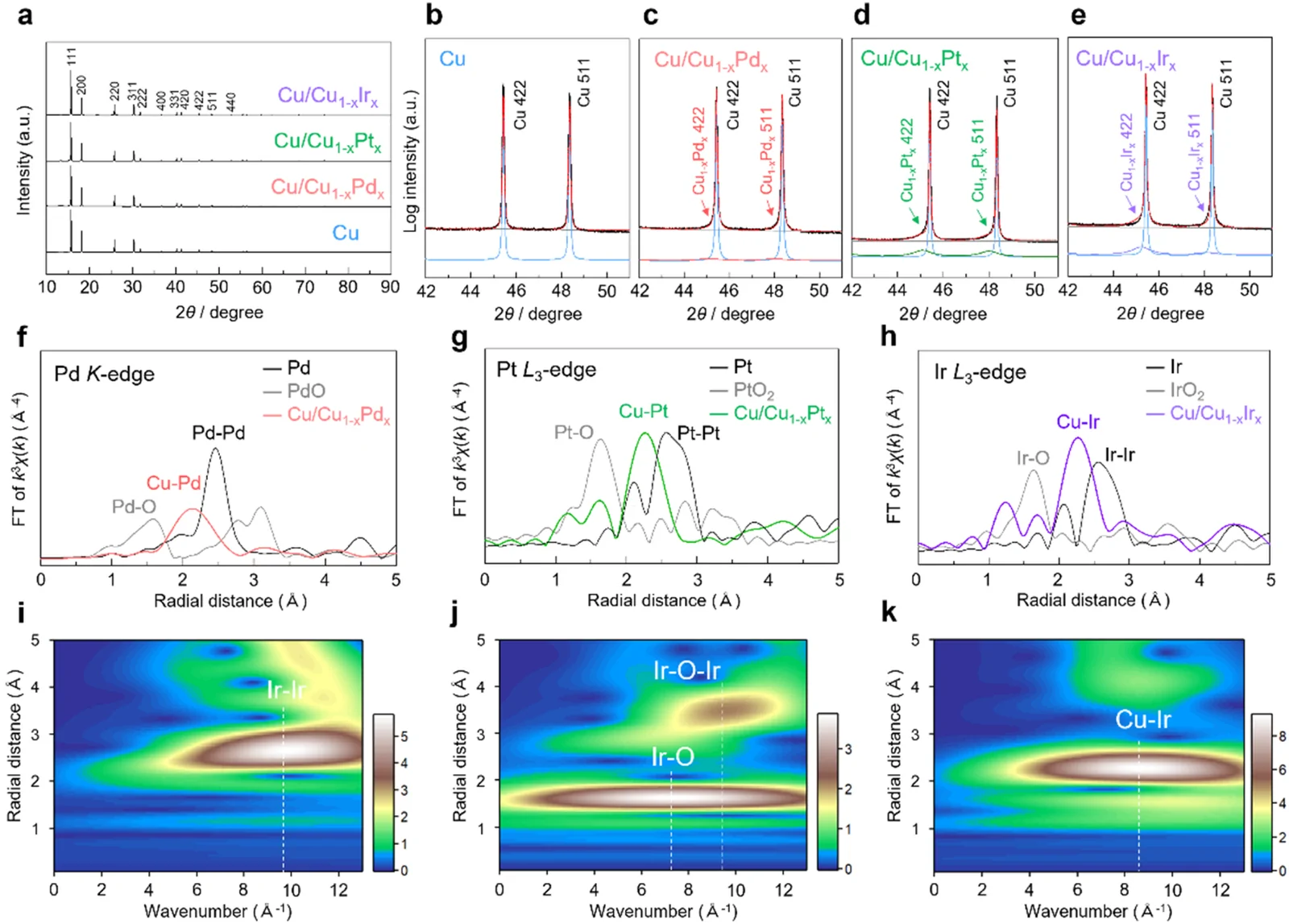
(a) XRD patterns and the expanded XRD patterns of (b) Cu, (c) Cu/Cu_1−*x*_Pd_*x*_, (d) Cu/Cu_1−*x*_Pt_*x*_ and (e) Cu/Cu_1−*x*_Ir_*x*_ NCs. EXAFS spectra at (f) the Pd K-edge of Cu/Cu_1−*x*_Pd_*x*_ NCs, (g) the Pt L_3_-edge of Cu/Cu_1−*x*_Pt_*x*_ NCs, and (h) the Ir L_3_-edge of Cu/Cu_1−*x*_Ir_*x*_ NCs. Ir L_3_-edge wavelet transform EXAFS of (i) Ir foil, (j) IrO_2_ powder and (k) Cu/Cu_1−*x*_Ir_*x*_ NCs. The colour bar represents the intensity range.

In the synchrotron X-ray diffraction (XRD) patterns of Cu/Cu_1−*x*_Pd_*x*_, Cu/Cu_1−*x*_Pt_*x*_ and Cu/Cu_1−*x*_Ir_*x*_ NCs, in addition to the diffraction peaks derived from fcc Cu NCs as the major component, broad fcc diffraction peaks appeared at angles lower than those of Cu NCs as the minor component (Fig. 2a–e and S9–S12). From Rietveld refinements, the lattice constants of the minor components were estimated to be 3.640(2), 3.641(1), and 3.631(1) Å for Cu/Cu_1−*x*_Pd_*x*_, Cu/Cu_1−*x*_Pt_*x*_ and Cu/Cu_1−*x*_Ir_*x*_ NCs, respectively (Fig. S9–S12). Assuming that the lattice constants follow Vegard's law, these minor components correspond to Cu_0.91_Pd_0.09_, Cu_0.92_Pt_0.08_, and Cu_0.92_Ir_0.08_ solid-solution alloys, respectively, which are highly consistent with the alloy compositions of Cu_1−*x*_M_*x*_ shell regions estimated from the STEM–EDX data (Fig. S6–S8 and Tables S1–S3). These results indicate the formation of the Cu_1−*x*_M_*x*_ solid-solution alloys on the surface of Cu NCs.

We performed X-ray absorption fine structure (XAFS) measurements to further characterize Cu/Cu_1−*x*_M_*x*_ (M = Pd, Pt, Ir). Fig. 2f–h show the Fourier-transformed (FT) Pd K-edge, Pt L_3_-edge, and Ir L_3_-edge extended X-ray absorption fine structure (EXAFS) spectra for Cu/Cu_1−*x*_Pd_*x*_, Cu/Cu_1−*x*_Pt_*x*_ and Cu/Cu_1−*x*_Ir_*x*_ NCs, respectively. In the *R*-space, prominent peaks were observed in the region of approximately 2.1–2.3 Å for Cu/Cu_1−*x*_Pd_*x*_ NCs and 2.2–2.3 Å for Cu/Cu_1−*x*_Pt_*x*_ and Cu/Cu_1−*x*_Ir_*x*_ NCs. These peaks are located between those of the corresponding standard metal and the oxide samples, indicating the formation of Cu–M bonds, while no typical peaks appear for the corresponding Pd–Pd, Pt–Pt, and Ir–Ir bonds. On the other hand, in the Cu K-edge FT-EXAFS spectra of Cu/Cu_1−*x*_M_*x*_, the prominent peak positions of the Cu–Cu contribution were similar to that of Cu NCs (2.2 Å) due to the strong influence of the Cu core and/or the Cu-rich alloy composition (approximately 90 at% Cu) of the shell (Fig. S13). The wavelet transform (WT) of Ir L_3_-edge EXAFS for Cu/Cu_1−*x*_Ir_*x*_ NCs was conducted to resolve the overlapping contributions from different scattering paths by simultaneously analyzing the *k*- and *R*-spaces. As shown in Fig. 2i and j, the WT contour plots of the Ir foil and IrO_2_ reference samples exhibited intensity maxima at 9.8 Å and 7.4 Å, corresponding to Ir–Ir and Ir–O scattering, respectively. In contrast, Cu/Cu_1−*x*_Ir_*x*_ NCs showed a single intensity maximum at 8.6 Å (Fig. 2k), which is attributed to Cu–Ir scattering. Similar WT features were observed for Cu/Cu_1−*x*_Pd_*x*_ and Cu/Cu_1−*x*_Pt_*x*_ NCs, confirming the solid-solution alloying in these systems as well (Fig. S14 and S15).

The electronic structure modification induced by the solid-solution alloying of Cu and M was investigated by X-ray photoelectron spectroscopy (XPS) measurements (Fig. S16–S18). The Pd 3d binding energy of Cu/Cu_1−*x*_Pd_*x*_ shifts positively relative to Pd powder, indicating that Pd is partially oxidized (Fig. S16). In contrast, the Pt 4f and Ir 4f binding energies of Cu/Cu_1−*x*_Pt_*x*_ (Fig. S17) and Cu/Cu_1−*x*_Ir_*x*_ NCs (Fig. S18) exhibit negative shifts relative to their bulk counterparts, indicating that Pt and Ir are partially reduced. Although the Cu 2p binding energy shifts are less distinguishable due to Cu surface oxidation upon exposure to air (Fig. S16–S18), the binding energy shifts of M suggest the occurrence of charge transfer from Pd to Cu in Cu/Cu_1−*x*_Pd_*x*_ NCs and from Cu to Pt or Ir in Cu/Cu_1−*x*_Pt_*x*_ and Cu/Cu_1−*x*_Ir_*x*_ NCs by solid-solution alloying, in agreement with density functional theory (DFT) calculations (Fig. S19 and Table S4).

The functions of solid-solution alloys can be continuously tuned by varying the compositions and combinations of their constituent elements.^3,26^ Most research on solid-solution alloy NPs has focused on combinations of elements that are miscible in the bulk phase under ambient conditions.^27^ Recently, non-equilibrium solid-solution alloy NPs, such as Cu–Ir,^28^ Cu–Ru,^29^ and Cu–Rh,^30^ have been reported due to advances in non-equilibrium solution-phase methods. However, achieving solid-solution alloying of immiscible combinations while retaining shape control remains a challenge in non-equilibrium synthesis methods that involve the rapid simultaneous reduction of metal precursors. In this study, we successfully synthesized shape-controlled solid-solution alloys of immiscible combinations using a galvanic replacement reaction. This approach includes not only the Cu–Ir system but also the Cu–Ru system (Fig. S20–S24), where the constituent elements are immiscible even above their melting points in the bulk phase, analogous to the immiscibility of oil and water.

**Fig. 3:**
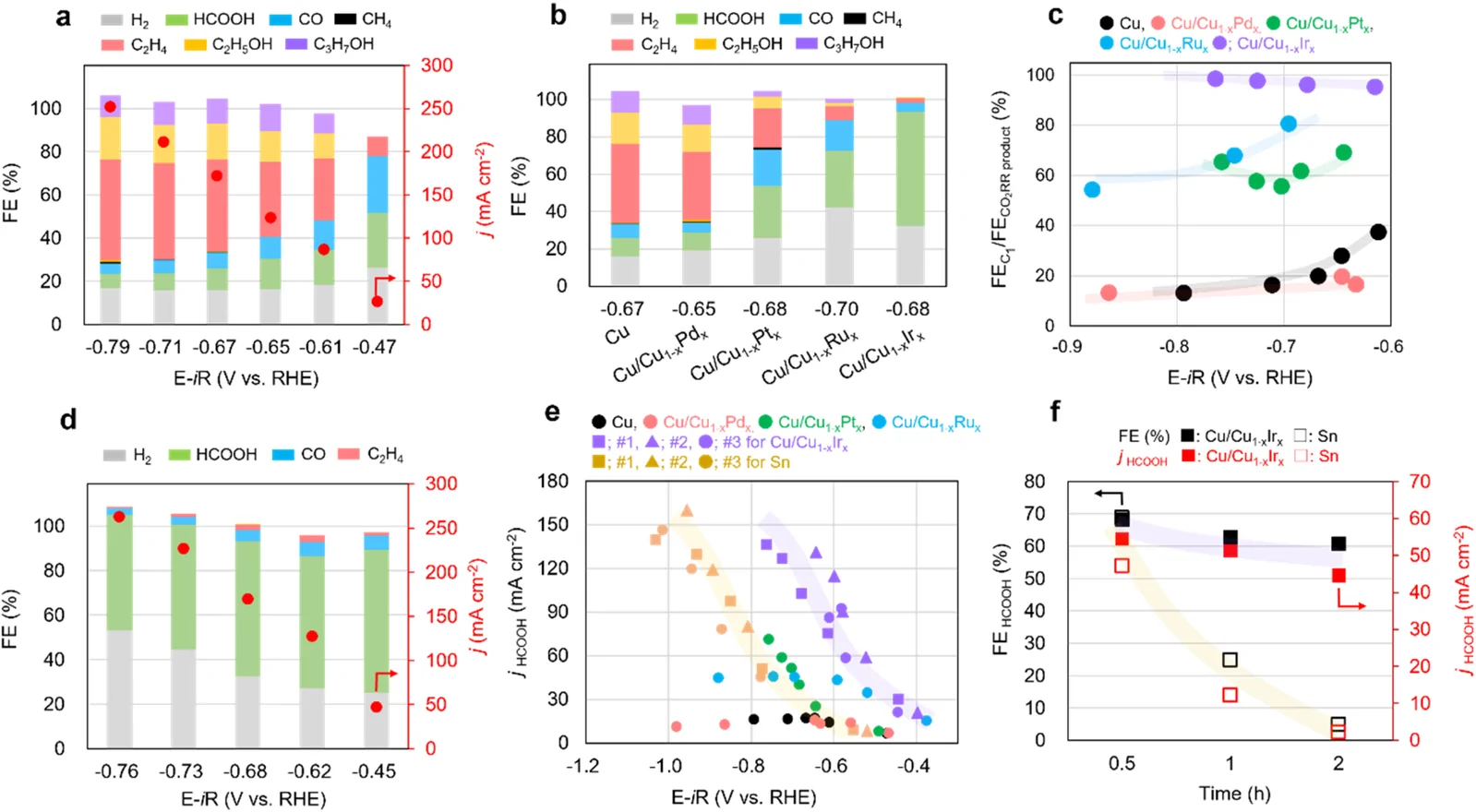
Faradaic efficiency of products on (a) Cu and (b) Cu/Cu_1−*x*_M_*x*_ NCs at similar applied potentials (−0.65 to −0.70 V *vs.* RHE). (c) C_1_/CO_2_RR product selectivity ratio on Cu/Cu_1−*x*_M_*x*_ NCs. (d) Faradaic efficiency of products on Cu/Cu_1−*x*_Ir_*x*_ NCs in a range of applied potentials. (e) Plot of partial current density of HCOOH *versus* applied potential on Cu NC, Sn NPs and Cu/Cu_1−*x*_M_*x*_. (f) Stability tests of Sn NPs and Cu/Cu_1−*x*_Ir_*x*_ NCs at 100 mA cm^−2^ current density for over 2 h.

The CO_2_RR performances of Cu/Cu_1−*x*_M_*x*_ NCs, (M = Pd, Pt, Ru, Ir) were evaluated in a three-electrode flow cell. We deposited the catalyst onto a carbon gas-diffusion electrode (GDE) *via* spray coating of a material ink and tested the samples in 1 M KOH electrolyte. As shown in Fig. 3a, Cu NCs exhibited high faradaic efficiency of C_2+_ products (FE_C_2+__) with applied potentials and achieved around 70% at −0.71 V *vs.* RHE which is consistent with previous reports.^12^ The CO_2_RR performance of Cu/Cu_1−*x*_M_*x*_ NCs was strongly dependent on the M species (Fig. 3b, d and S25–S27), and replacing Cu with only 1 at% of Pd, Pt, Ru, and Ir atoms in sequence resulted in a gradual decrease in the selectivity for C_2+_ products and an increase in the selectivity for C_1_ components, particularly HCOOH at similar applied potentials (−0.65 to −0.70 V *vs.* RHE) (Fig. 3b). Remarkably, Cu/Cu_1−*x*_Ir_*x*_ NCs exhibited a high C_1_/CO_2_RR product ratio across the applied potential range of −0.6 to −0.9 V *vs.* RHE (Fig. 3c) with high HCOOH selectivity (Fig. 3d). To date, most alloying strategies to improve CO_2_RR performance involve combining a primary product-forming metal with a secondary one – for example, Cu-based alloys for C_2+_ products^31^ or p-block-based alloys for HCOOH.^32^ Here we demonstrate successful product switching from C_2_H_4_ to HCOOH by solid-solution surface alloying of Cu with HER-active Ir. This result motivated a comparative study with a representative Sn catalyst for CO_2_ to HCOOH conversion.^9,32^ Although the Sn NPs exhibited slightly higher selectivity for HCOOH than the Cu/Cu_1−*x*_Ir_*x*_ NCs (Fig. S28), the different three Cu/Cu_1−*x*_Ir_*x*_ NCs provided higher partial current densities over the entire potential range, together with a ∼0.3 V lower overpotential than the Sn NPs (Fig. 3e and S29). In addition, stability tests were performed at a conversion rate of −100 mA cm^−2^ for Sn NPs and Cu/Cu_1−*x*_Ir_*x*_ NCs, respectively (Fig. 3f). The Sn NPs exhibited a rapid decrease in both current density and HCOOH selectivity over time, accompanied by an increase in the HER. This degradation results from the leaching of Sn from the GDE, caused by its dissolution into the electrolyte through the reaction with strongly alkaline KOH. In contrast, Cu/Cu_1−*x*_Ir_*x*_ NCs retained both high HCOOH selectivity and current density. Notably, whereas p-block element-based catalysts are typically unstable under alkaline conditions, Cu/Cu_1−*x*_Ir_*x*_ NCs exhibit robust stability and activity.

**Fig. 4:**
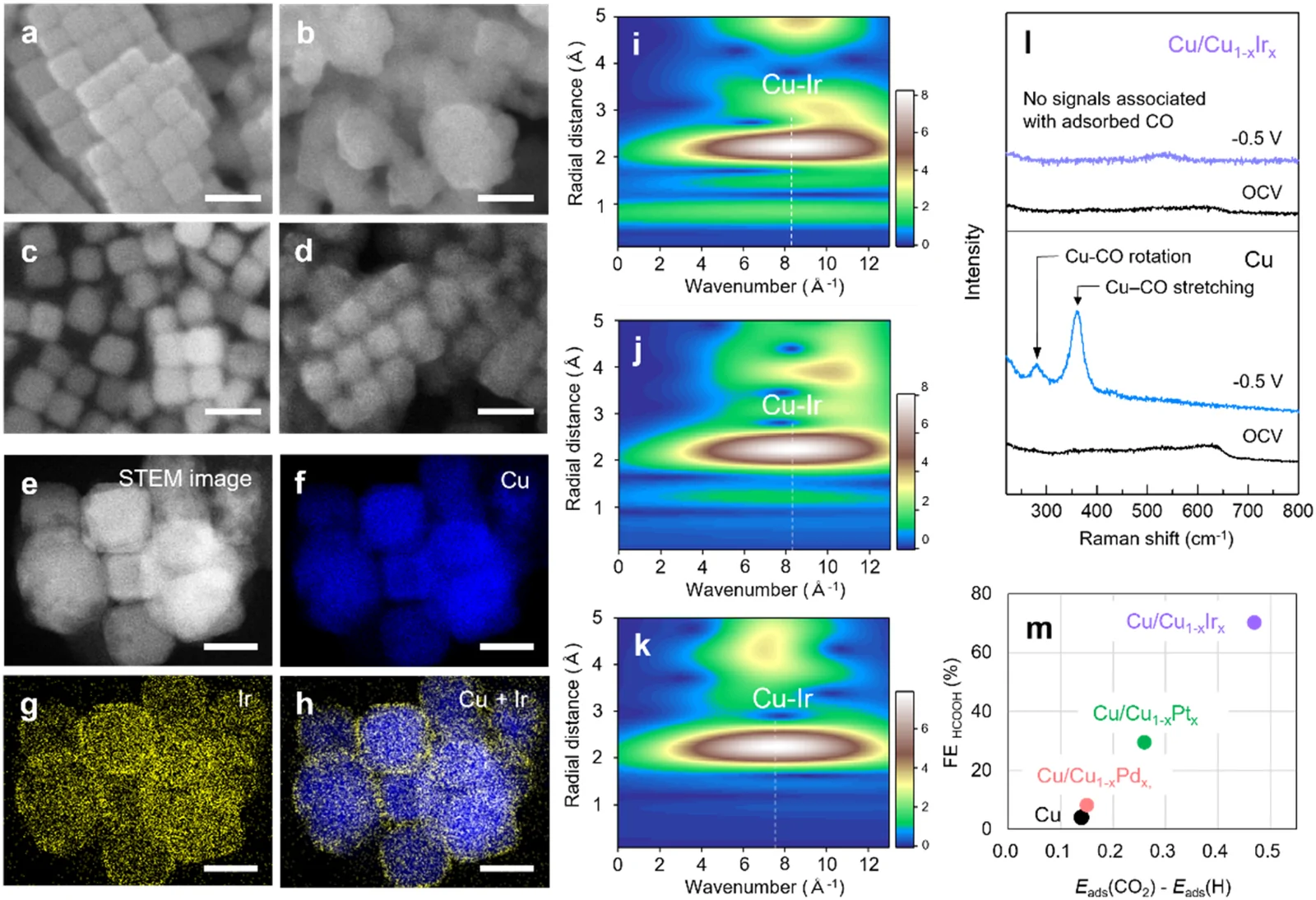
SEM images of (a and b) Cu and (c and d) Cu/Cu_1−*x*_Ir_*x*_ NCs (a and c) before and (b and d) after the CO_2_RR. Scale bars, 100 nm. (e–h) STEM images and EDX maps of Cu/Cu_1−*x*_Ir_*x*_ NCs after the CO_2_RR. Scale bars, 50 nm. Ir L_3_-edge wavelet transform EXAFS of Cu/Cu_1−*x*_Ir_*x*_ NCs at (i) OCV, open-circuit voltage, (j) −0.5 V and (k) OCV after −0.5 V. (l) *In situ* Raman spectra recorded at OCV and −0.5 V *vs.* RHE for Cu and Cu/Cu_1−*x*_Ir_*x*_ NCs, and (m) the differential adsorption energies (CO_2_*vs.* H*) on Cu NC and the Cu/Cu_1−*x*_M_*x*_ NCs (M = Pd, Pt, Ir).

To gain insight into the mechanism underlying the shift in product selectivity from C_2_H_4_ to HCOOH, and the remarkable activity and stability in HCOOH production on Cu/Cu_1−*x*_Ir_*x*_ NCs, we first investigated their structure after CO_2_RR testing. SEM images before and after the CO_2_RR indicated that the cubic morphology of Cu NCs degraded substantially, accompanied by particle growth from 50 nm to 200 nm (Fig. 4a, b and S30). In contrast, the morphology and particle size of Cu/Cu_1−*x*_Ir_*x*_ NCs were relatively retained (Fig. 4c and d). STEM–EDX mapping indicated the preservation of the Cu–Ir solid-solution surface alloy (Fig. 4e–h) after the CO_2_RR. Furthermore, *in situ* FT-EXAFS analyses at the Ir L_3_-edge and Cu K-edge, along with its WT, revealed that the Ir–Cu (2.2–2.3 Å) and Cu–Cu (2.2 Å) bond lengths and a single intensity maximum at approximately *k* = 8 Å^−1^ were retained during the CO_2_RR (Fig. S31, S32 and 4i–k), demonstrating the persistence of the Cu–Ir solid-solution alloy structure on cubic Cu NCs under reaction conditions.

As CO_2_RR performance is highly sensitive to catalyst features such as facet orientation, particle size, and alloying state, strategies to preserve these structural parameters under reaction conditions are crucial. Although a few studies have explored approaches such as hetero-element incorporation through alloying^33,34^ or encapsulation with alumina shells,^35^ strategies to stabilize catalyst structures during the CO_2_RR remain scarce. In particular, retaining the size and morphology of Cu NCs at high current densities (>100 mA cm^−2^) is rarely addressed, since (100)-oriented Cu NCs typically undergo drastic reconstruction into crystalline domains even at low current densities (<10 mA cm^−2^).^25^ Here, we show the first demonstration that Ir incorporation effectively stabilizes both the size and morphology of Cu NCs, thereby contributing to the sustained stability in HCOOH production (Fig. 3f).

Given that Ir NPs produced only H_2_*via* the HER (Fig. S33) and that a physical mixture of Ir NPs and Cu NCs resulted in low HCOOH selectivity (Fig. S34), the shift in product selectivity from C_2_H_4_ to HCOOH observed for Cu/Cu_1−*x*_Ir_*x*_ NCs arises from atomic-level surface alloying of Cu and Ir. To investigate CO_2_RR pathways, *in situ* Raman spectroscopy was conducted on the different catalysts. For Cu NCs, distinct peaks appeared at ∼280 and 360 cm^−1^ at an applied potential of −0.5 V *vs.* RHE (Fig. 4l), corresponding to the restricted rotation of adsorbed CO and Cu–CO stretching, respectively.^36^ *CO is the key intermediate that has been proposed for C–C coupling towards C_2+_ or further hydrogenation to C_1_ species such as CH_4_.^23^ Similar peaks were observed on Cu/Cu_1−*x*_Pd_*x*_ NCs (Fig. S35), which also exhibit C_2+_ product selectivity at the same potential (Fig. S25). In contrast, no CO*-related peaks were detected on Cu/Cu_1−*x*_Ir_*x*_ or Cu/Cu_1−*x*_Pt_*x*_ NCs at −0.5 V *vs.* RHE (Fig. 4l and S36). These results suggest that alloying Cu with Ir alters the reaction pathway from C_2+_ formation toward HCOOH production through a CO*-free pathway.^23^

From SEM measurements on Cu/Cu_1−*x*_Pd_*x*_ and Cu/Cu_1−*x*_Pt_*x*_ NCs after the CO_2_RR (Fig. S37), Cu/Cu_1−*x*_Pd_*x*_ exhibited morphological degradation, similar to that observed for Cu NCs after the CO_2_RR. In contrast, Cu/Cu_1−*x*_Pt_*x*_ NCs retained their cubic shape, similar to Cu/Cu_1−*x*_Ir_*x*_ NCs. We also confirmed this trend by STEM–EDX mapping analyses (Fig. S38 and S39). Furthermore, *in situ* XAFS measurements demonstrated that the Cu–Pt solid-solution alloy structure was preserved on the cubic Cu NCs under reaction conditions (Fig. S40). Recently, it has been reported that the degradation of cubic Cu nanocrystals into nanograins is driven by the adsorption of CO intermediates, followed by the formation of Cu carbonyl species at an applied potential.^24^ Therefore, the distinct HCOOH formation pathway *via* a CO*-free mechanism on Cu/Cu_1−*x*_Pt_*x*_ and Cu/Cu_1−*x*_Ir_*x*_ NCs is considered to play an important role in stabilizing both the size and morphology of the cubic Cu NCs during the CO_2_RR.

COOH* and HCOO* are critical intermediates in determining the product distribution in the CO_2_RR, where COOH*—formed by the reaction of chemisorbed CO_2_* with water-derived protons—leads to CO and C_2+_ products *via* an asymmetric coupling between CO* and another intermediate such as CH_2_*,^37^ while HCOO*—formed by the direct reaction of physisorbed CO_2_ with surface H*—drives HCOOH formation,^38,39^ and high H* coverage on the catalyst surface promotes HCOOH formation.^40^ To elucidate the origin of HCOOH formation on Cu/Cu_1−*x*_Ir_*x*_ NCs, we evaluated the adsorption energies of key intermediates (COOH*, HCOO* and H*) and CO_2_ on Cu NCs and Cu/Cu_1−*x*_M_*x*_ NCs (M = Pd, Pt, Ir) using DFT calculations. The Cu/Cu_1−*x*_M_*x*_ NCs were modelled using a Cu(100) slab, where three surface Cu layers were partially replaced with M atoms (Fig. S19). The results show that COOH* adsorption is more stable than HCOO* adsorption on Cu/Cu_1−*x*_Ir_*x*_ or Cu/Cu_1−*x*_Pt_*x*_ NCs, whereas HCOO* is more stable on Cu NCs and Cu/Cu_1−*x*_Pd_*x*_ NCs (Fig. S41–S45 and Table S6). This stabilization of the COOH* intermediate implies that CO or C_2_ formation is more favourable on Cu/Cu_1−*x*_Ir_*x*_ or Cu/Cu_1−*x*_Pt_*x*_ surfaces than on Cu NCs and Cu/Cu_1−*x*_Pd_*x*_ NCs, in contrast to the experimental results (Fig. 3b). These discrepancies highlight the importance of considering the adsorption of CO_2_ and H* prior to intermediate formation. The *H adsorption energy becomes stronger from Cu (−0.39 eV) and Cu/Cu_1−*x*_Pd_*x*_ (−0.45 eV) to Cu/Cu_1−*x*_Pt_*x*_ (−0.57 eV) and Cu/Cu_1−*x*_Ir_*x*_ (−0.85 eV), while the CO_2_ adsorption energies range from −0.25 to −0.37 eV over Cu_1−*x*_M_*x*_ surfaces (Table S5). As shown in Fig. 4m, the differential adsorption energies (CO_2_*vs.* H*) strongly correlate with FE for HCOOH, suggesting that strong H* adsorption on Cu/Cu_1−*x*_Ir_*x*_ NCs plays a key role in promoting HCOO* formation *via* a direct reaction with physisorbed CO_2_. While Sn in p-block metals requires a high overpotential for H* supply, isolated HER-active Ir atoms on Cu/Cu_1−*x*_Ir_*x*_ NCs efficiently provide H*, thereby decreasing the overpotential and enhancing catalytic activity compared with Sn NPs (Fig. 3e).

## Conclusions

In this work, we have designed and developed Cu/Cu_1−*x*_M_*x*_ NCs, M = Pd, Pt, Ru, Ir, for the first comprehensive investigation of CO_2_ reduction on Cu-based NCs with HER-active platinum group metals. The Cu/Cu_1−*x*_M_*x*_ NCs were successfully synthesized through a galvanic replacement reaction, with Cu_1−*x*_M_*x*_ solid-solution surface alloying confirmed by XRD, EXAFS and HAADF-STEM. Among them, Cu/Cu_1−*x*_Ir_*x*_ NCs achieved highly selective HCOOH production at an overpotential ∼0.3 V lower than that of conventional Sn catalysts. The active H* adsorbed on Cu/Cu_1−*x*_Ir_*x*_ NCs plays a key role in promoting HCOOH production with the low overpotential. Furthermore, Cu/Cu_1−*x*_Ir_*x*_ NCs demonstrate high operational stability, retaining high activity without degradation, in contrast to the rapid deactivation of Sn catalysts. The alloying with Ir effectively stabilized both the size and morphology of Cu NCs. These findings present a versatile strategy for tuning reaction pathways through isolated heteroatom interfaces, offering broad applicability to other elemental combinations and electrocatalytic reactions.

## Author contributions

H. K. and M. Y. designed the research. S. H. performed the synthesis, most of the structural characterization studies, and electrochemical tests. H. K., A. A., T. M., M. U., T. G. N. and M. D. performed the XAS measurement and analysed the EXAFS and XANES data under the supervision of T. S. and H. S. H. K. and S. H. collected and analysed the STEM data under the supervision of T. Y. and Y. M. H. K., M. D. and T. G. N. collected and analysed the synchrotron X-ray diffraction data under the supervision of K. K. T. S. collected and analysed the SAXS data. Y. S. and T. I. conducted DFT calculations. All authors discussed the results and commented on the manuscript.

## Conflicts of interest

There are no conflicts to declare.

## Supplementary Material

SC-017-D6SC02600A-s001

## Data Availability

All relevant data are presented in the main text and supplementary information (SI). Supplementary information is available. See DOI: https://doi.org/10.1039/d6sc02600a.
