# Supplementary material for: Solid-solution surface alloying of Cu nanocubes with platinum-group metals: pathway switching and catalyst stabilization in CO2 reduction
Source: Chem Sci. 2026 May 26;17(26):12852–9. doi: 10.1039/d6sc02600a (PMC13203979; doi:10.1039/d6sc02600a)
Supplement: SC-017-D6SC02600A-s001 [file SC-017-D6SC02600A-s001.pdf]

Supplementary Information (SI) for Chemical Science.
This journal is © The Royal Society of Chemistry 2026

Supporting Information

Solid-Solution Surface Alloying of Cu Nanocubes with Platinum-Group Metals: Pathway Switching and Catalyst Stabilization in CO_2 Reduction

Hirokazu Kobayashi,*[a] Sachie Hikino,[a] Akihiko Anzai,[b],[+] Takahiro Matsuu,[c] Mahiru Umeno,[c] Tomohiro G. Noguchi,[b] Masaki Donoshita,[b] Tomokazu Yamamoto,[d] Yasukazu Murakami,[d,e] Kenichi Kato,[f] Takeharu Sugiyama,[g] Hiroyuki Setoyama,[h] Tetsuroh Shirasawa,[i] Yuya Shimohata,[j] Takayoshi Ishimoto,[j] Miho Yamauchi*[a,b,c,k,l]

a. Research Center for Negative Emissions Technologies, Kyushu University, 744 Motooka, Nishi-ku, Fukuoka 819-0395, Japan.

b. Institute for Materials Chemistry and Engineering (IMCE), Kyushu University, 744 Motooka, Nishi-ku, Fukuoka 819-0395, Japan

c. Department of Chemistry, Faculty of Science, Kyushu University, 744 Motooka, Nishi-ku, Fukuoka 819-0395, Japan

d. The Ultramicroscopy Research Center, Kyushu University, Motooka 744, Nishi-ku, Fukuoka 819-0395, Japan

e. Department of Applied Quantum Physics and Nuclear Engineering, Kyushu University, Motooka 744, Nishi-ku, Fukuoka 819-0395, Japan

f. RIKEN SPring-8 Center, 1-1-1 Kouto, Sayo-cho, Sayo-gun, Hyogo 679-5148, Japan

g. Research Center for Synchrotron Light Applications, Kyushu University, 6-1 Kasuga Park, Kasuga-shi, Fukuoka 816-8580, Japan

h. Beamline Group, SAGA Light Source,6-1 Kasuga Park, Kasuga-shi, Fukuoka 816-8580, Japan

i. National Institute of Advanced Industrial Science and Technology (AIST), Research Institute for Measurement and Analysis Instrumentation, 1-1-1 Higashi, Tsukuba, Ibaraki 305-8565, Japan

j. Smart Innovation Program, Graduate School of Advanced Science and Engineering, Hiroshima University,1-4-1 Kagamiyama, Higashihiroshima, Hiroshima 739-8527, Japan

k. International Institute for Carbon-Neutral Energy Research (WPI-I2CNER), Kyushu University,744 Motooka, Nishi-ku, Fukuoka 819-0395, Japan

l. Advanced Institute for Materials Research (WPI-AIMR), Tohoku University, 2-1-1 Katahira, Aoba-ku, Sendai, 980-8577 Japan

† Current address: Institute for Catalysis Hokkaido University, N-21, W-10, Sapporo 001-0021, Japan

E-mail: kobayashi@k-nets.kyushu-u.ac.jp, yamauchi@ms.ifoc.kyushu-u.ac.jp

Experimental Procedures

Chemicals. Copper(I) bromide (CuBr, 95.0%), ethanol (EtOH, 99.5%), hexane (96.0%), oleylamine (OLAM, technical grade), potassium hydroxide (KOH), platinum(II) acetylacetonate ($Pt(acac)_2$, 98%) and palladium(II) acetylacetonate ($Pd(acac)_2$, 98%) were purchased from Fujifilm Wako Pure Chemical Corporation. Tri-n-octylphosphine oxide (TOPO, >95.0%), iridium(III) acetylacetonate ($Ir(acac)_3$, 99.9%) and ruthenium(III) acetylacetonate ($Ru(acac)_3$, 99.9%) were purchased from Tokyo Chemical Industry Co., Ltd.. Sn nanoparticles with an average diameter of 50 nm were purchased from EM Japan Co., Ltd. All chemicals were used without further purification.

Synthesis of Cu NCs. Cu NCs were synthesized following a previously reported method with slight modifications.[1] In a typical procedure, 7.2 mmol of TOPO and 35 ml of OLAM were added to a three-neck flask. The solution was then degassed under vacuum for 30 minutes to remove dissolved oxygen and moisture. After switching to an argon atmosphere, 1.5 mmol of CuBr was added to the mixture. Subsequently, the solution was first heated to 80 °C and maintained for 15 minutes, followed by a temperature ramp at 10 °C/min to 260 °C, where it was held for 1 hour. After cooling to room temperature, the precipitate was centrifuged and washed four times with ethanol and hexane.

Synthesis of $Cu/Cu_{1-x}M_x$ NCs. 7.2 mmol of TOPO and 35 ml of OLAM were added to a three-neck flask. The solution was then degassed under vacuum for 30 minutes to remove dissolved oxygen and moisture. After switching to an argon atmosphere, 1.5 mmol of CuBr was added to the mixture. Subsequently, the solution was first heated to 80 °C and maintained for 15 minutes, followed by a temperature ramp at 10 °C/min to 260 °C, where it was held for 1 hour. For the synthesis of $Cu/Cu_{1-x}Pd_x$ NCs, the solution was cooled to 180 °C, and 4.1 μmol $Pd(acac)_2$ OAm solution was injected, followed by incubation at this temperature for 30 min. After cooling to room temperature, the precipitate was centrifuged, washed four times with hexane and ethanol. $Cu/Cu_{1-x}Pt_x$, $Cu/Cu_{1-x}Ir_x$, and $Cu/Cu_{1-x}Ru_x$ NCs were synthesized using similar procedures to those for $Cu/Cu_{1-x}Pd_x$ NCs, with the following adjustments: the precursors were changed to 12.7 μmol $Pt(acac)_2$, 27.8 μmol $Ir(acac)_3$, and 50.2 μmol/ml $Ru(acac)_3$ OAm solution, respectively, and the incubation temperatures and times were modified to 240 °C for 60 min for $Cu/Cu_{1-x}Pt_x$ NCs, 220 °C for 360 min for $Cu/Cu_{1-x}Ir_x$ NCs, and 240 °C for 60 min for $Cu/Cu_{1-x}Ru_x$ NCs.

Electrode preparation. The catalyst ink was prepared by adding catalyst into a toluene solution of 1 mL, and then ultrasonically treated it for 10 min. A carbon gas-diffusion electrode was prepared via spray coating of the material ink on a typical carbon paper gas diffusion layer (Sigracet 39 BB). Catalyst loadings of 0.5 mg/cm² were used, and 0.2 mg/cm² was selected for the stability tests.

Characterizations. Transmission electron microscope (TEM) images were captured with a JEM-2100 HC instrument operated at 200 kV acceleration voltage. The samples for TEM were dispersed in toluene, dropped onto a carbon-coated copper grid, and dried by exposure to ambient conditions for 24 h. Scanning electron microscopy (SEM) images and X-ray energy dispersive spectroscopy (EDX) mapping were taken on a JEOL JSM-7900F microscope or JEOL JSM-IT100 microscope. SAXS measurements were performed at beamline BL-4C, Photon Factory, High Energy Acceleration Research Organization (KEK), using 11 keV X-rays. Scattered X-rays were recorded with a PILATUS-100K pixel array area detector (DECTRIS Ltd.) positioned 873 mm from the sample. The SAXS profile was fitted with an isotropic core-shell cube form factor[2], assuming a Cu core with a Cu/Ir shell (1 nm thick, 6 at% Ir), to determine the edge length distribution modeled with the Schultz–Zimm function. Atomic-resolution high-angle annular dark-field scanning TEM (HAADF-STEM) images and EDX mapping were taken on a JEOL ARM 200F STEM instrument operated at a 200 kV accelerating voltage. Synchrotron powder X-ray diffraction (XRD) measurements were performed at the Super Photon Ring (SPring-8) BL44B2 beam line.[3] The synchrotron XRD patterns of the samples sealed in a glass capillary were measured with wavelengths of 0.5699 Å. The XRD patterns of the samples were also measured using a Panalytical X'Pert Pro X-ray powder diffractometer with Cu-Kα radiation. Rietveld analyses were performed using a Topas software package (Bruker AXS Inc., Billerica, MA, USA, version 5). X-ray absorption fine structure (XAFS) spectra were collected at the BL06 and BL07 beam lines of Kyushu Synchrotron Light Research Center (SAGA-LS, Japan). XAFS spectra were recorded in the total electron yield mode using a four-element silicon drift detector (SDD). The reference spectra of Cu, Cu_2O, CuO, Pd, PdO, Pt, PtO_2, Ir, IrO_2 Ru and RuO_2 were recorded in the transmission mode. The powders of the reference samples were well mixed with boron nitride (BN) and then pressed into a pellet for measurement. XAFS data analyses were performed with the Athena software package. X-ray photoelectron spectroscopy (XPS) studies were performed on a PHI5000 VersaProbeII (ULVAC-PHI) using Al Kα radiation (1486.6 eV). Binding energies in XPS spectra were corrected by referring to a C 1s binding energy of the carbon atoms of the ligand in the specimens at 284.5 eV.

DFT calculations. All calculations were performed using the plane-wave DFT method implemented in the Vienna ab initio simulation package (VASP 6.2.0)[4-6]. The Perdew-Burke-Ernzerhof parametrization[7] within the generalized gradient approximation was used as the exchange-correlation functional, with the projector-augmented wave method[8]. Spin-polarized calculations were consistently applied, with the plane-wave cutoff energy of 400 eV. The bulk structure of Cu was optimised using a 16 × 16 × 16 k-points based on the Monkhorst-Pack method[9]. Using the optimized bulk structure, a six-layered Cu(100) slab model with a vacuum layer of 15 A was constructed. CuM(100) surfaces (M = Pd, Pt, and Ir) were modelled based on the Cu(100) slab by substituting and dispersing metal atoms within the top three layers (Supplementary Fig. 20). There three layers consist of 40 Cu atoms and 14 M atoms, with M atoms accounting for approximately 26 % of the surface composition. The overall concentration of M

atoms in the entire slab model is around 13 %. Geometry optimization of both the Cu(100) and CuM(100) surfaces was performed only to the top three layers using a 4 × 4 × 1 k-points. Additionally, all calculations involving the metal surfaces employed the DFT-D3 method[10].

Electrochemical measurements. Electrochemical measurements were performed using a three-electrode system in an electrochemical flow cell setup configuration. The cathode in the flow cell has a geometric area of 1 cm^2, which is used for all current density calculations. A flow rate of 4 ml min^{-1} was employed for introducing 1 M KOH aqueous solution into the cathode chamber, while a flow rate of 1 mL min^{-1} was used for introducing 1 M KOH aqueous solution into the anode chamber, respectively by two pumps. The cathode camber and anode chamber were separated by a Nafion 117 cation exchange membrane. Pure CO_2 gas (99.99%) was continuously supplied to the gas chamber of the flow cell at a total flow rate of 15 mL min^{-1}. CO_2RR performance was investigated by using constant-potential electrolysis, i.e., chronoamperometry while purging CO_2 into the catholyte during the whole electrochemical test. The potentials versus the Hg/HgO reference electrode were converted to values versus the reversible hydrogen electrode (RHE) using the following equation. All measurements were iR-corrected.

E (V vs. RHE) = E (V vs. Hg/HgO) + 0.098 V + 0.0591 V × pH

Gas products were analyzed using on-line gas chromatography (Micro GC Fusion®, Inficon, Bad Ragaz, Switzerland) equipped with a Molsieve 5A column and a plot Q column coupled with a thermal conductivity detector (TCD). Liquid products were analysed using a high-performance liquid chromatograph (HPLC, LC-20AD, Shimadzu) equipped with a refractive-index detector (RID-10A, Shimadzu). The Faradaic efficiency (FE) of products in the electrochemical CO_2 reduction experiments is calculated using the following equation:

$$FE\,(\%) = \frac{n \times z \times F}{Q} \times 100$$

where n is the number of moles of product, and z represents the number of electrons required for the formation of product (e.g., z = 2 for CO, formic acid, and H_2; z = 8 for CH_4 and CH_3COOH; z = 12 for C_2H_4 and C_2H_5OH). F is the Faraday constant (96,485 C mol^{-1} of electrons). Q is the amount of charge passed during the electrolysis. For the gas products, n was calculated as follows:

$$n_{gas}\,(mol) = p_0 \times x \times v \times t \times R \times T$$

where x is the volume fraction of gas product; P_0 is atmospheric pressure (1 atm); v is the CO_2 flow rate (L min^{-1}); t is electrolysis time; R is the ideal gas constant (0.08205 L atm mol^{-1} K^{-1}); T is room temperature (298 K).

In-situ XAFS measurements. In-situ XAFS experiments were performed under the same conditions as the electrochemical tests, using a modified flow cell with an opening in the gas chamber sealed by Kapton tape. In-situ Cu K- and Ir L_3-edge XAFS spectra were measured at the Kyushu University

beamline BL06 of Kyushu Synchrotron Light Research Center (SAGA-LS, Japan) with an electron storage ring operating at the energy of 1.4 GeV. The energy range of this light source (bending magnet) is 2.1–23 keV. A silicon (111) double-crystal monochromator was used to collect the incident X-ray beam. The typical photon flux is 10^{10} photons per second. In-situ XAFS spectra were recorded in the fluorescence mode using a four-element silicon drift detector (SDD). Data processing was performed using Athena and Artemis, which are included in the Ifeffit package.[11] Wavelet transforms were computed using a software module developed by Marina Chukalina (IMT-RAS) and Harald Funke (IRC-FZR),[12,13] employing a Morlet wavelet function with κσ values set to 15.

In-situ Raman spectroscopy measurements. Raman spectra were acquired using an STR Raman spectrometer (AIRIX Corp, Japan) equipped with a 785 nm excitation laser. The laser operated at a power of 1 mW, and observations were conducted using a 20× magnification objective. Prior to collecting each Raman spectrum, CO_2RR was pre-stabilized for 120 seconds, ensuring stable reaction conditions for data acquisition. A custom-made electrochemical Raman flow cell was employed. This flow cell design featured a thin electrolyte layer of approximately 3 mm, enabling direct laser irradiation of the catalyst surface through a light window. Within this setup, a prepared GDE served as the working electrode. The counter electrode was a coiled Pt wire (100 × 0.5 mm^2), and Ag/AgCl (1.0 M NaCl) was utilized as the reference electrode. Nafion-117 was positioned to separate the catholyte and anolyte chambers. A flow rate of 4 ml min^{-1} was employed for introducing 1 M KOH aqueous solution into the cathode chamber, while a flow rate of 10 mL min^{-1} was used for introducing 1 M KOH aqueous solution into the anode chamber, respectively by two pumps. Simultaneously, CO_2 was continuously introduced into the gas chamber at a controlled flow rate of 50 mL min^{-1}.

References

1. De Gregorio, G. L.; Burdyny, T.; Loiudice, A.; Iyengar, P.; Smith, W. A.; Buonsanti, R. Facet-Dependent Selectivity of Cu Catalysts in Electrochemical CO2 Reduction at Commercially Viable Current Densities. *ACS Catal*. **2020**, *10*, 4854–4862.
2. Jones, M. R.; Macfarlane, R. J.; Lee, B.; Zhang, J.; Young, K. L.; Senesi, A. J.; Mirkin, C. A. DNA-Nanoparticle Superlattices Formed from Anisotropic Building Blocks. *Nat. Mater*. **2010**, *9*, 913–917.
3. Kato, K.; Tanaka, Y.; Yamauchi, M.; Ohara, K.; Hatsui, T. A Statistical Approach to Correct X-ray Response Nonuniformity in Microstrip Detectors for High Accuracy and High-Resolution Total-Scattering Measurements. *J. Synchrotron Radiat*. **2019**, *26*, 762–773.
4. Kresse, G.; Hafner, J. Ab Initio Molecular Dynamics for Liquid Metals. *Phys. Rev. B* **1993**, *47*, 558–561.
5. Kresse, G.; Furthmüller, J. Efficient Iterative Schemes for Ab Initio Total-Energy Calculations Using a Plane-Wave Basis Set. *Phys. Rev. B* **1996**, *54*, 11169–11186.

6. Kresse, G.; Furthmüller, J. Efficiency of Ab Initio Total Energy Calculations for Metals and Semiconductors Using a Plane-Wave Basis Set. *Comput. Mater. Sci*. **1996**, *6*, 15–50.
7. Perdew, J. P.; Burke, K.; Ernzerhof, M. Generalized Gradient Approximation Made Simple. *Phys. Rev. Lett*. **1996**, *77*, 3865–3868.
8. Blöchl, P. E. Projector Augmented-Wave Method. *Phys. Rev. B* **1994**, *50*, 17953–17979.
9. Monkhorst, H. J.; Pack, J. D. Special Points for Brillouin-Zone Integrations. *Phys. Rev. B* **1976**, *13*, 5188–5192.
10. Grimme, S.; Antony, J.; Ehrlich, S.; Kreig, H. A Consistent and Accurate Ab Initio Parametrization of Density Functional Dispersion Correction (DFT-D) for the 94 Elements H–Pu. *J. Chem. Phys*. **2010**, *132*, 154104.
11. Ravel, B.; Newville, M. ATHENA, ARTEMIS, HEPHAESTUS: Data Analysis for X-ray Absorption Spectroscopy Using IFEFFIT. *J. Synchrotron Radiat*. **2005**, *12*, 537–541.
12. Funke, H.; Scheinost, A. C.; Chukalina, M. Wavelet Analysis of Extended X-ray Absorption Fine Structure Data. *Phys. Rev. B* **2005**, *71*, 094110.
13. Funke, H.; Chukalina, M.; Scheinost, A. C. A New FEFF-Based Wavelet for EXAFS Data Analysis. *J. Synchrotron Radiat*. **2007**, *14*, 426–432.

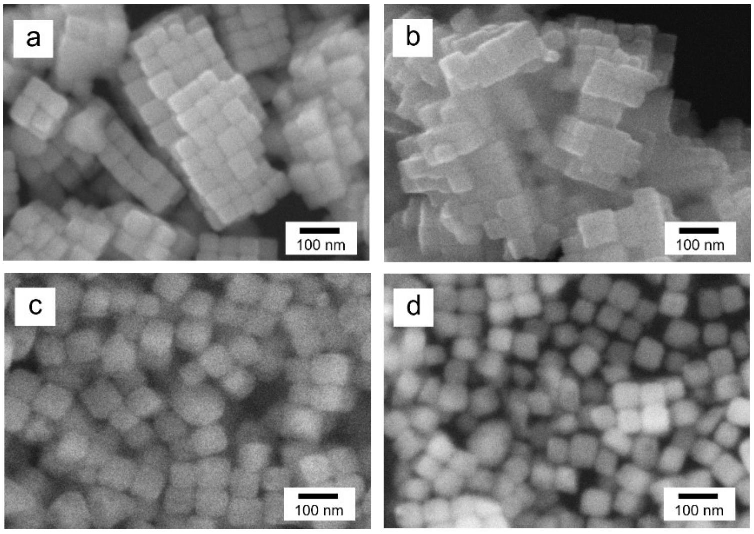

Figure S1. SEM images of (a) Cu NCs, (b) Cu/$Cu_{1-x}Pd_x$, (c) Cu/$Cu_{1-x}Pt_x$ and (d) Cu/$Cu_{1-x}Ir_x$ NCs.

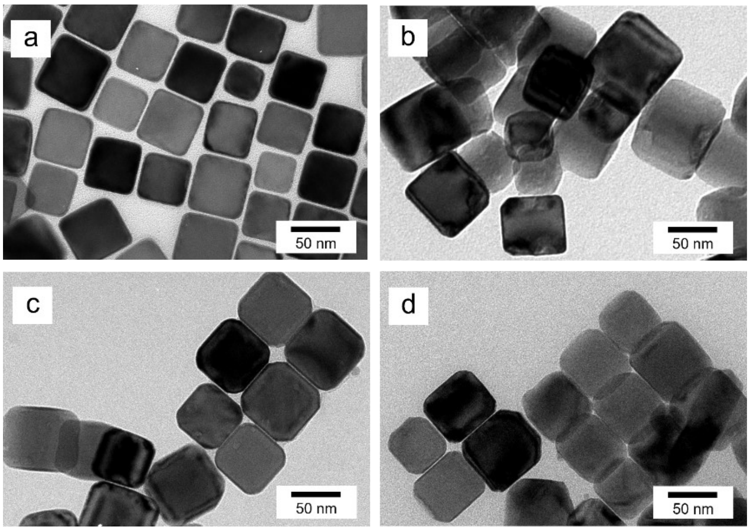

Figure S2. TEM images of (a) Cu NCs, (b) Cu/$Cu_{1-x}Pd_x$, (c) Cu/$Cu_{1-x}Pt_x$ and (d) Cu/$Cu_{1-x}Ir_x$ NCs.

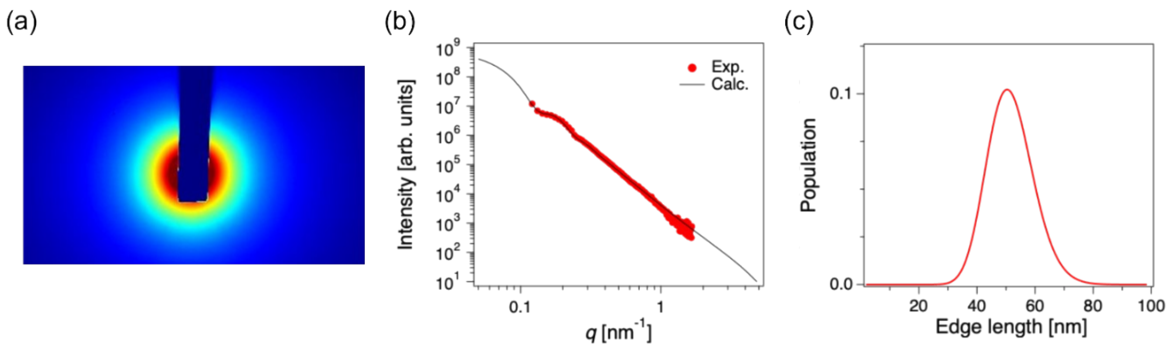

Figure S3. Small-angle X-ray scattering (SAXS) analysis of $Cu/Cu_{1-x}Ir_x$ NCs: (a) 2D detector image, (b) 1D SAXS profile obtained by azimuthal integration, and (c) edge length distribution derived from fitting with a core–shell cube model using the Schultz–Zimm function.

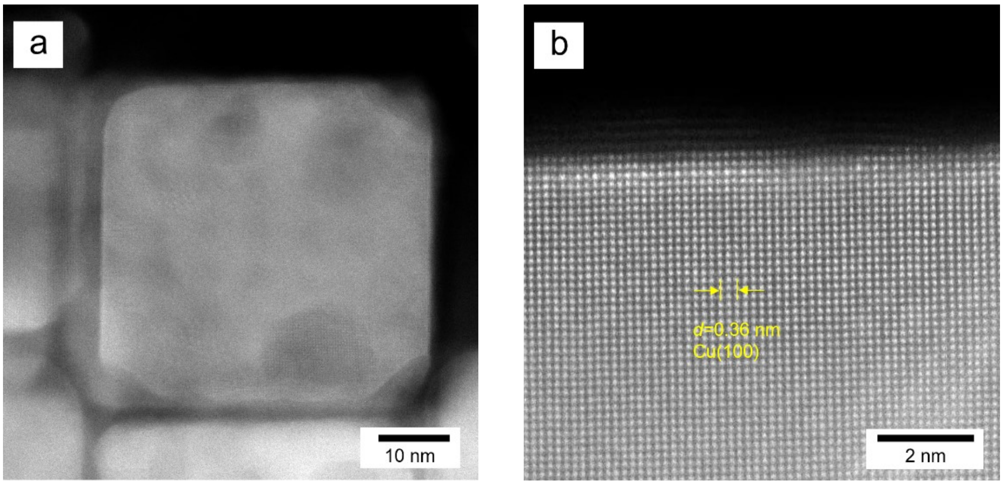

Figure S4. (a) HAADF-STEM and (b) atomic-resolution HAADF-STEM images of $Cu/Cu_{1-x}Pd_x$ NCs. The thickness of the $Cu_{1-x}Pd_x$ shell was 4-5 atomic layers.

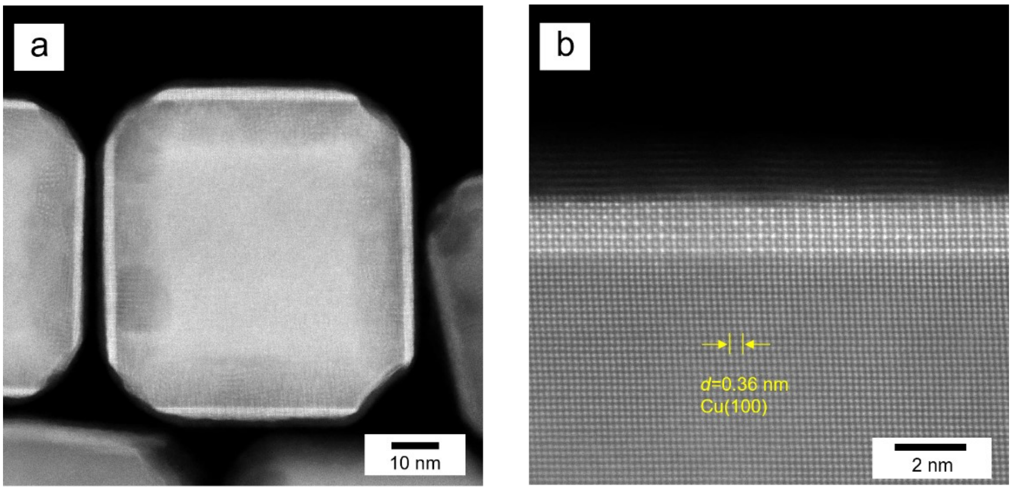

Figure S5. (a) HAADF-STEM and (b) atomic-resolution HAADF-STEM images of Cu/$Cu_{1-x}Pt_x$ NCs. The thickness of the $Cu_{1-x}Pt_x$ shell was 8 atomic layers.

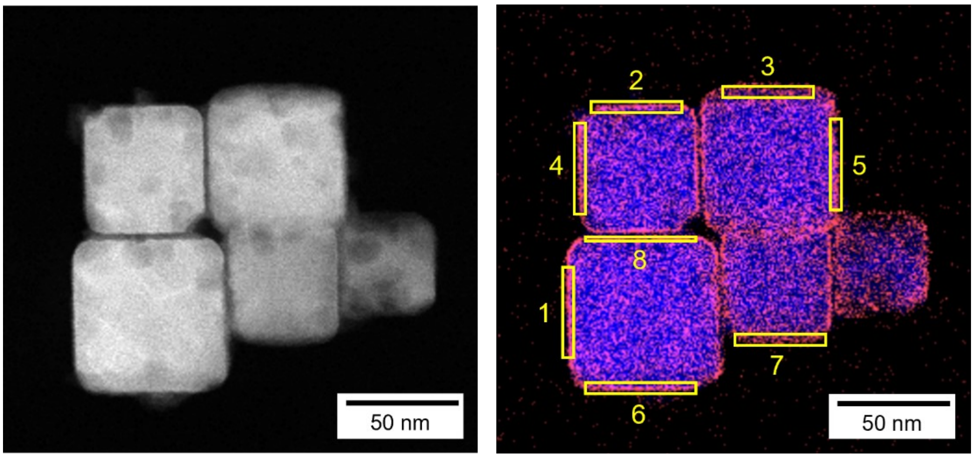

Figure S6. (a) HAADF-STEM image and (b) EDX map of Cu/$Cu_{1-x}Pd_x$ NCs.

Table S1. Composition of Cu and Pd in $Cu_{1-x}Pd_x$ shell regions estimated from STEM-EDX maps in Figure S6.

	**Cu (at%)**	**Pd (at%)**
Area 1	87.6±2.6	12.4±1.3
Area 2	89.4±3.4	10.7±1.6
Area 3	93.7±3.9	6.4±1.0
Area 4	87.6±2.6	12.4±1.3
Area 5	93.9±3.5	6.2±0.9
Area 6	92.6±3.1	7.4±1.4
Area 7	91.6±4.0	8.4±1.2
Area 8	89.0±3.3	11.0±1.5
**Average**	**90.7±3.3**	**9.4±1.3**

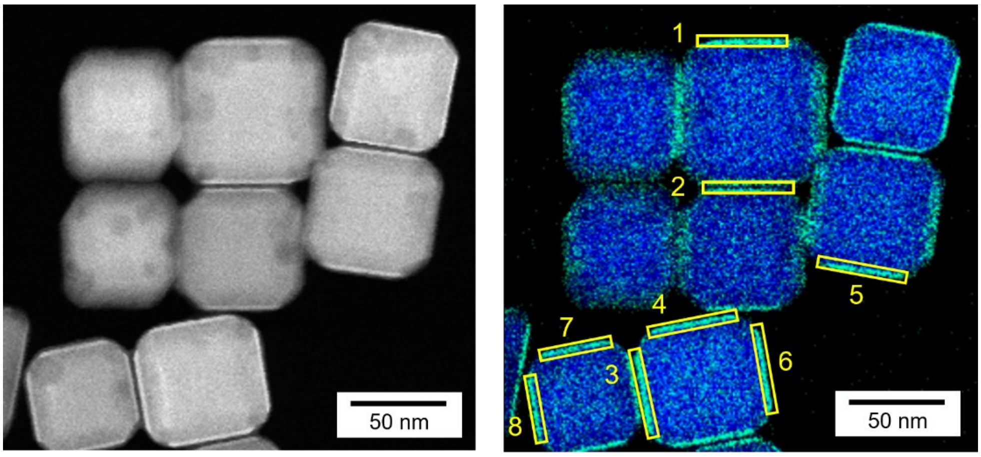

Figure S7. (a) HAADF-STEM image and (b) EDX map of $Cu/Cu_{1-x}Pt_x$ NCs.

Table S2. Composition of Cu and Pt in $Cu_{1-x}Pt_x$ shell regions estimated from STEM-EDX maps in Figure S7.

	**Cu (at%)**	**Pt (at%)**
Area 1	92.4±2.9	7.7±0.9
Area 2	92.1±3.0	7.9±1.0
Area 3	91.1±3.4	8.9±1.2
Area 4	92.1±3.1	7.9±1.0
Area 5	93.1±3.4	6.9±1.0
Area 6	93.0±3.1	7.0±1.0
Area 7	92.1±5.5	7.9±1.7
Area 8	92.1±4.0	7.9±1.2
**Average**	**92.3±3.6**	**7.8±1.1**

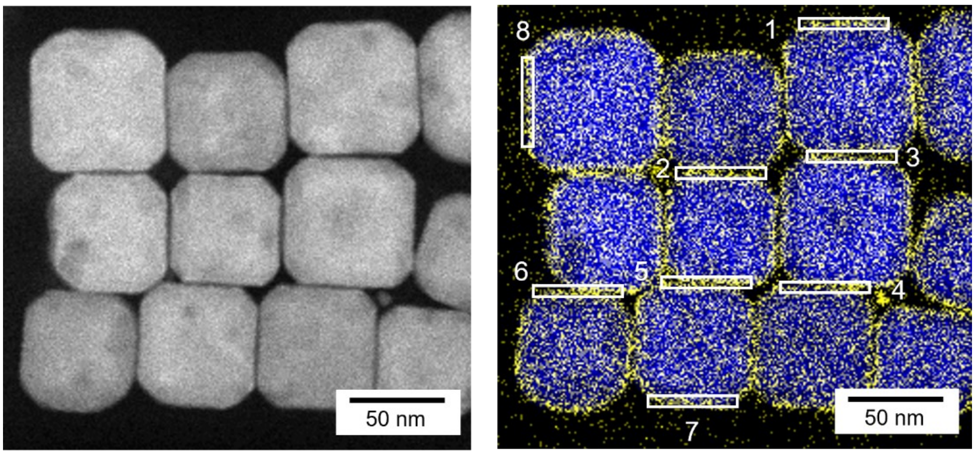

Figure S8. (a) HAADF-STEM image and (b) EDX map of $Cu/Cu_{1-x}Ir_x$ NCs.

Table S3. Composition of Cu and Ir in $Cu_{1-x}Ir_x$ shell regions estimated from STEM-EDX maps in Figure S8.

	**Cu (at%)**	**Ir (at%)**
Area 1	92.3±6.1	7.7±2.6
Area 2	93.3±4.0	6.7±1.3
Area 3	95.2±4.8	4.8±1.6
Area 4	94.8±4.0	5.2±1.2
Area 5	95.2±3.6	4.8±1.2
Area 6	94.6±4.3	5.4±1.5
Area 7	93.8±5.7	6.2±1.8
Area 8	92.8±4.0	7.2±1.6
**Average**	**94.0±4.6**	**6.0±1.6**

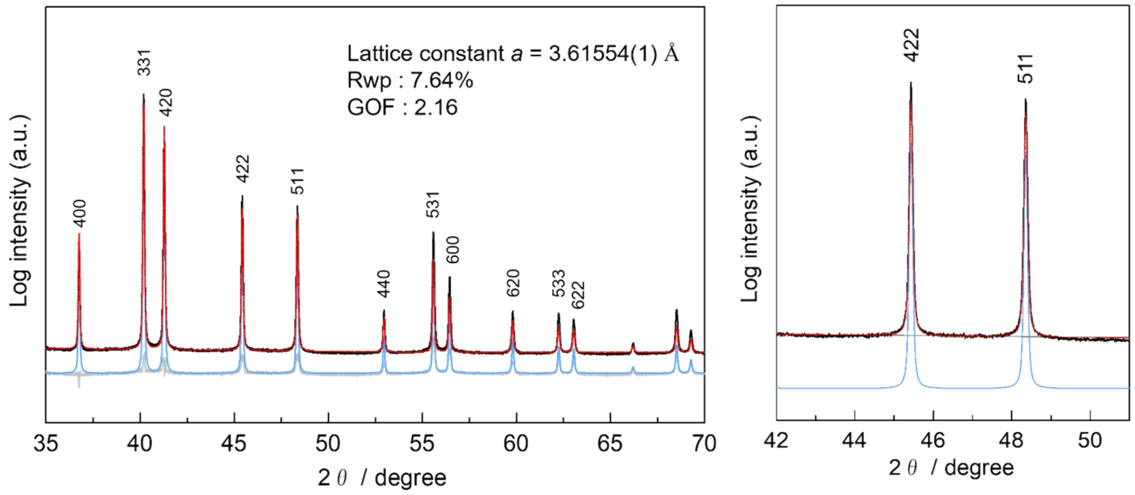

Figure S9. Synchrotron XRD pattern of Cu NCs (black line) at 303 K, and calculated pattern (red dots). The bottom lines show the difference profile (grey) and the fitting curves of the Cu component (blue). The radiation wavelength was 0.5699 Å.

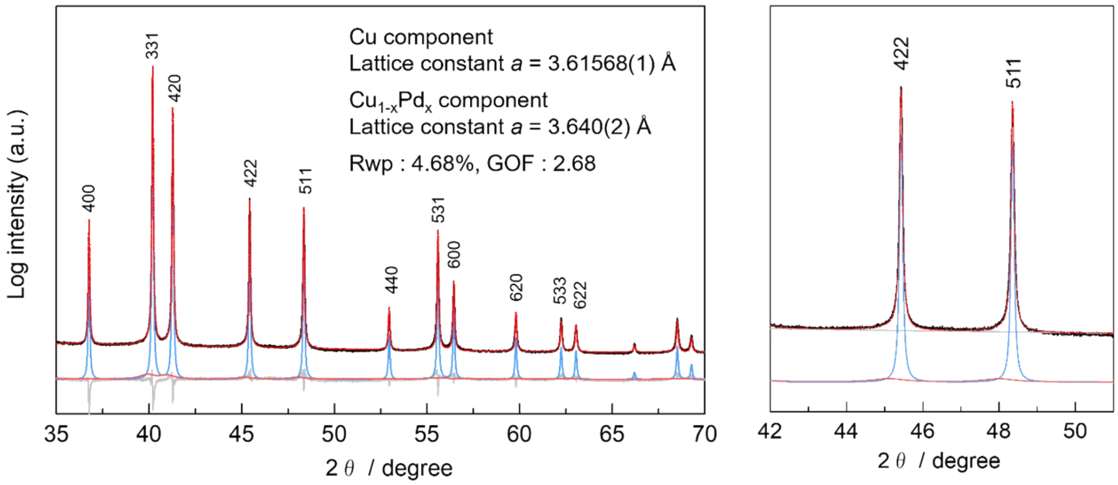

Figure S10. Synchrotron XRD pattern of $Cu/Cu_{1-x}Pd_x$ NCs (black line) at 303 K, and calculated pattern (red dots). The bottom lines show the difference profile (grey) and the fitting curves of the Cu (blue) and $Cu_{1-x}Pd_x$ (pink) components. The radiation wavelength was 0.5699 Å.

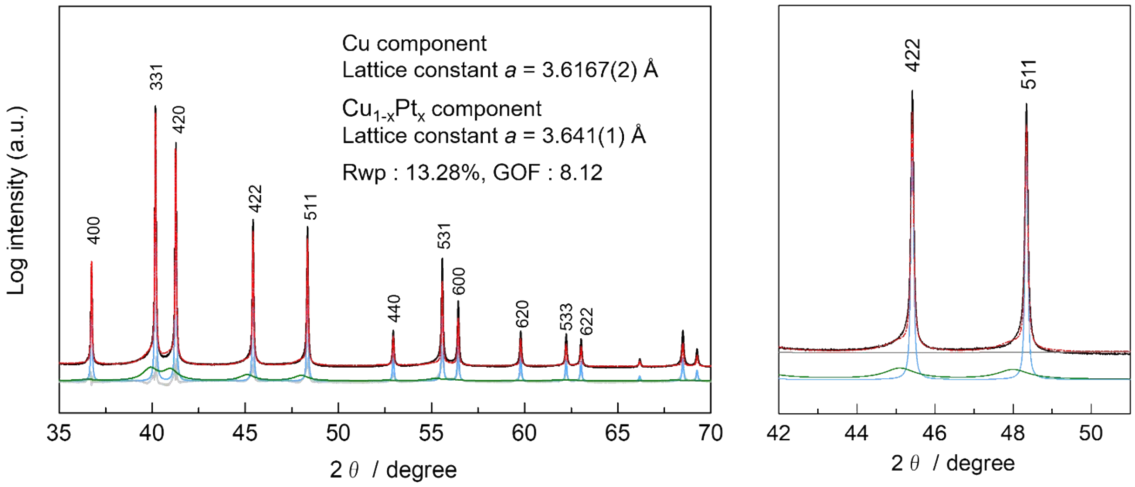

Figure S11. Synchrotron XRD pattern of $Cu/Cu_{1-x}Pt_x$ NCs (black line) at 303 K, and calculated pattern (red dots). The bottom lines show the difference profile (grey) and the fitting curves of the Cu (blue) and $Cu_{1-x}Pt_x$ (green) components. The radiation wavelength was 0.5699 Å.

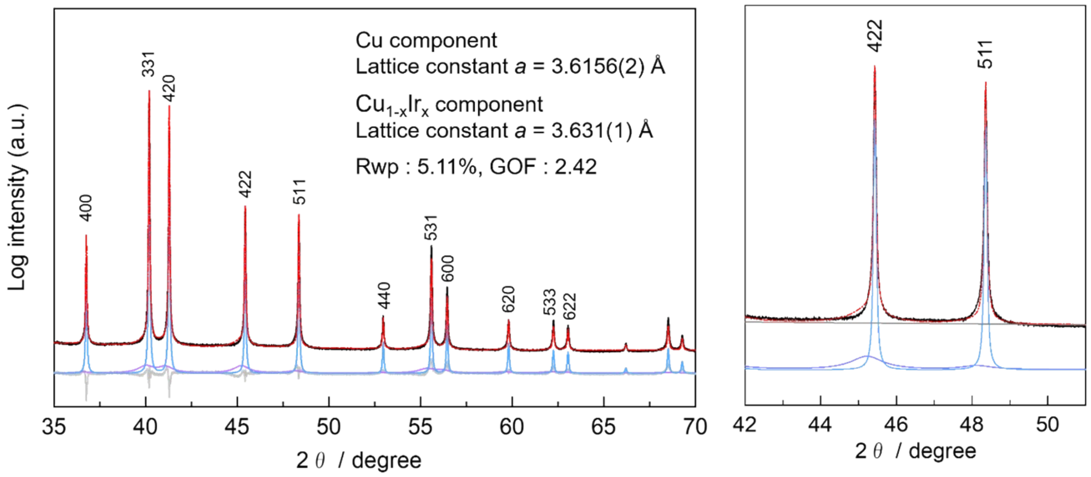

Figure S12. Synchrotron XRD pattern of $Cu/Cu_{1-x}Ir_x$ NCs (black line) at 303 K, and calculated pattern (red dots). The bottom lines show the difference profile (grey) and the fitting curves of the Cu (blue) and $Cu_{1-x}Ir_x$ (purple) components. The radiation wavelength was 0.5699 Å.

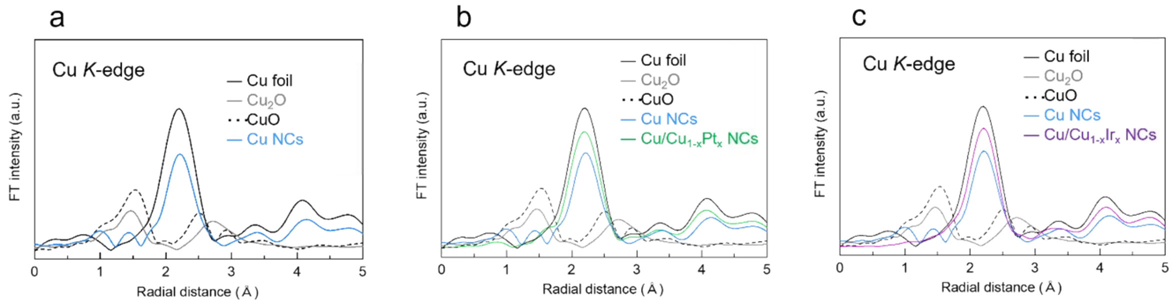

Figure S13. EXAFS spectra at the Cu *K*-edge of (a) Cu, (b) Cu/$Cu_{1-x}Pt_x$ and (c) Cu/$Cu_{1-x}Ir_x$ NCs.

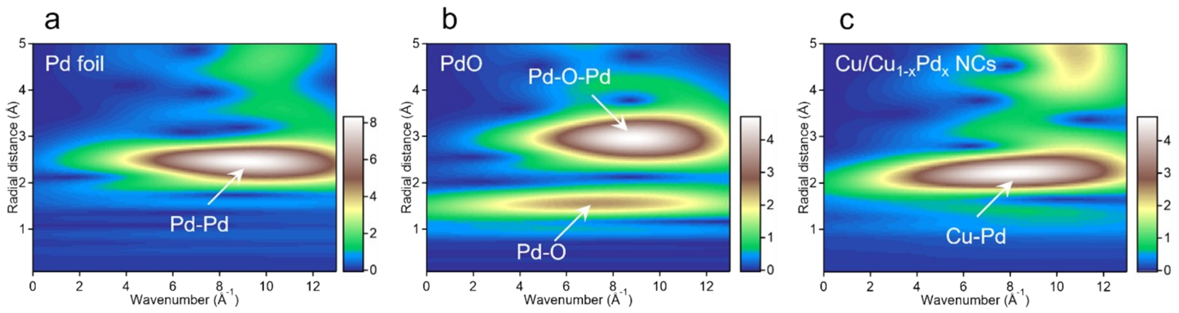

Figure S14. Pd *K*-edge wavelet transform EXAFS of (a) Pd foil, (b) PdO powder and (c) Cu/$Cu_{1-x}Pd_x$ NCs. The colour bar represents the intensity range.

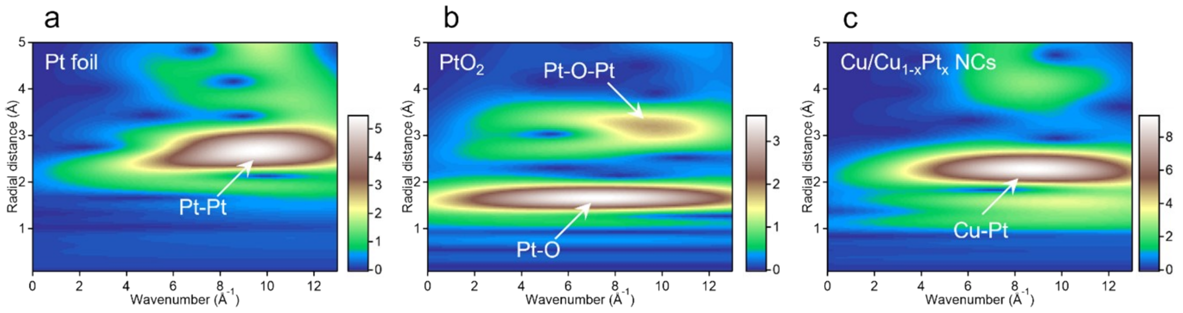

Figure S15. Pt L_3-edge wavelet transform EXAFS of (a) Pt foil, (b) PtO_2 powder and (c) Cu/$Cu_{1-x}Pt_x$ NCs. The colour bar represents the intensity range.

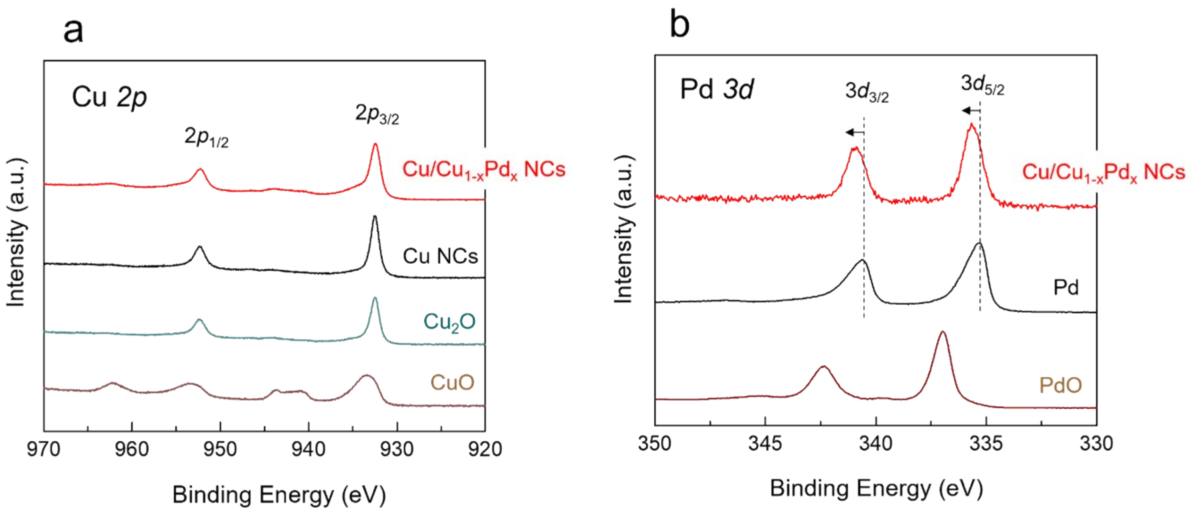

Figure S16. XPS spectra of (a) Cu 2*p* and (b) Pd 3*d* for $Cu/Cu_{1-x}Pd_x$ NCs.

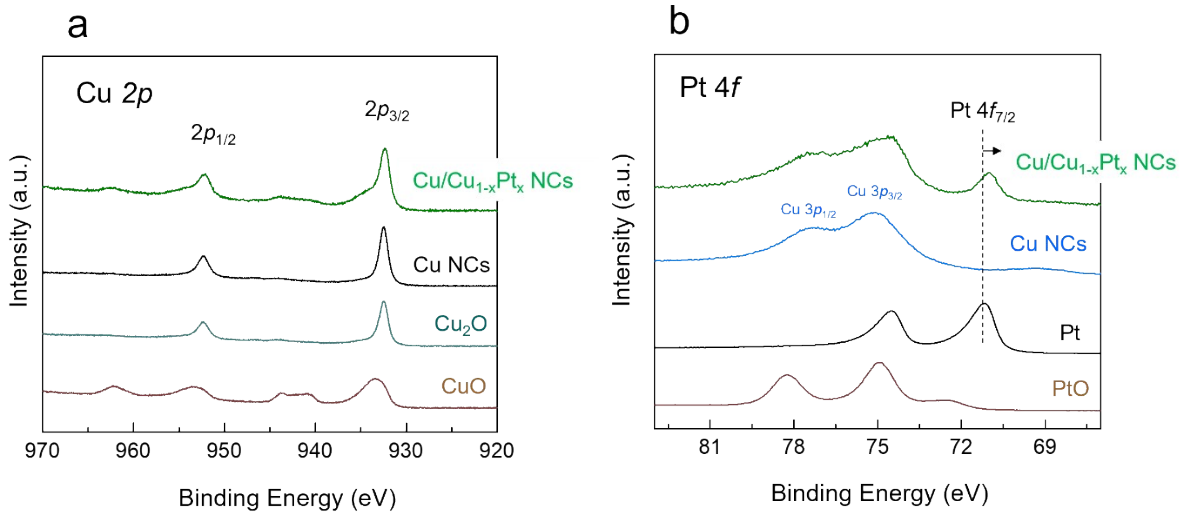

Figure S17. XPS spectra of (a) Cu 2*p* and (b) Pt 4*f* for $Cu/Cu_{1-x}Pt_x$ NCs.

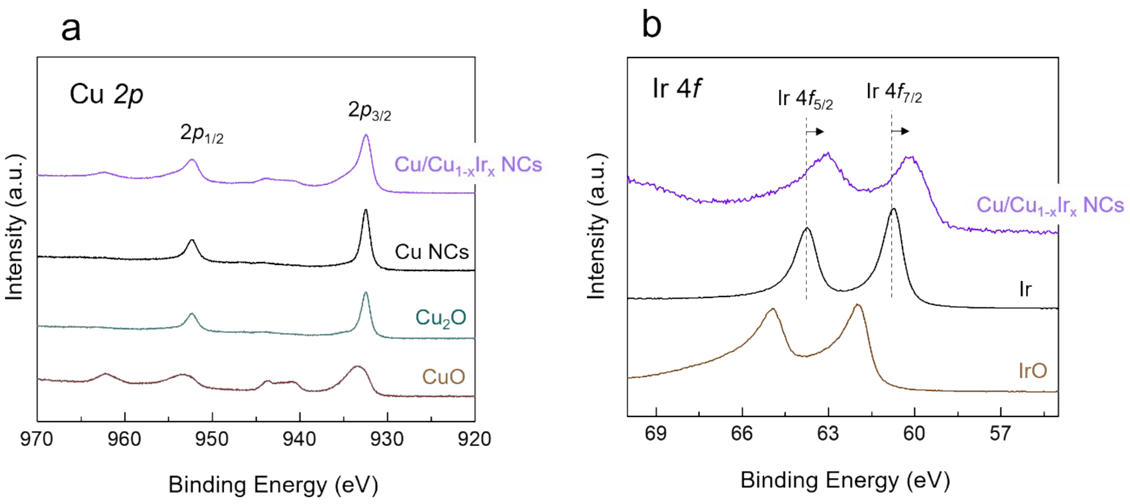

Figure S18. XPS spectra of (a) Cu 2*p* and (b) Ir 4*f* for $Cu/Cu_{1-x}Ir_x$ NCs.

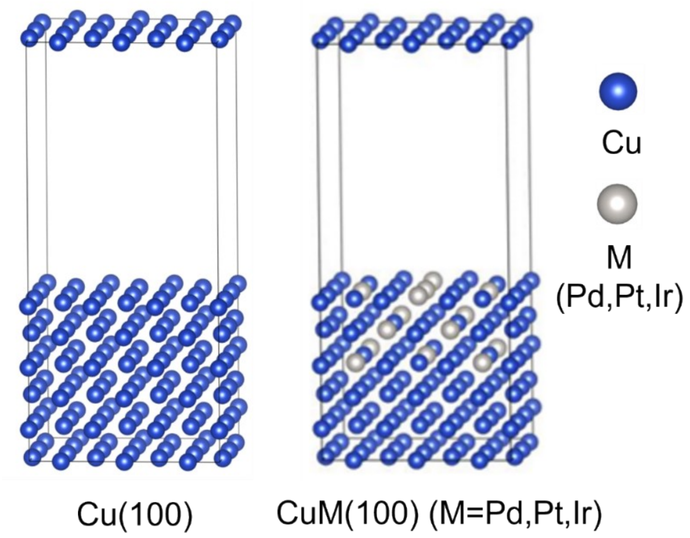

Figure S19. Surface models of $Cu/Cu_{1-x}M_x$ NCs (M=Pd, Pt, Ir).

Table S4. Average of atomic charges for each atom and total charge of surface layer in Cu(100) and CuM(100) (M = Pd, Pt, and Ir) models by DFT calculations.

	Cu atom	M atom	Total
Cu	-0.001	-	-0.026
CuPd	-0.320	0.130	-0.191
CuPt	0.787	-1.115	-0.329
CuIr	1.008	-1.370	-0.362

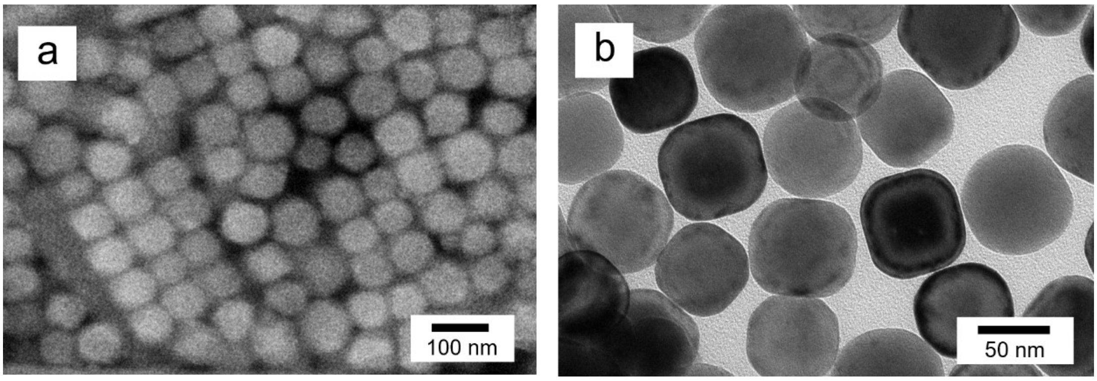

Figure S20. (a) SEM and (b) TEM images of $Cu/Cu_{1-x}Ru_x$ NCs. From SEM-EDX analysis, the atomic percentages of Ru included in the $Cu/Cu_{1-x}Ru_x$ NCs was calculated to be 0.9 %.

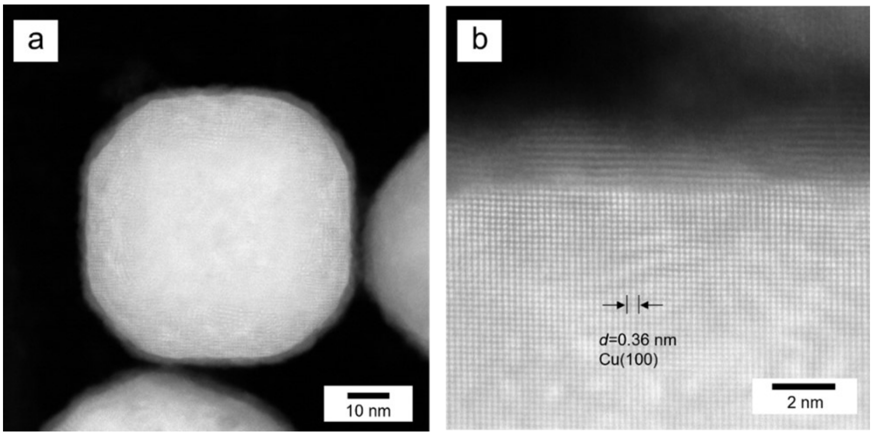

Figure S21. (a) HAADF-STEM and (b) atomic-resolution HAADF-STEM images of $Cu/Cu_{1-x}Ru_x$ NCs.

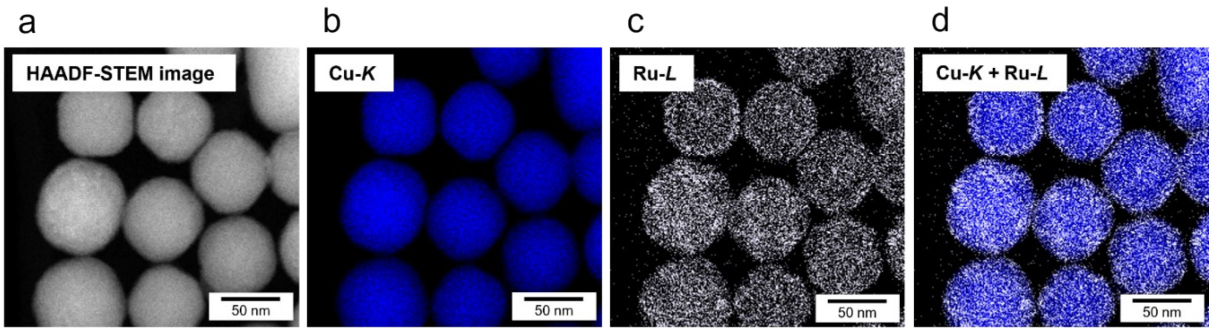

Figure S22. (a) HAADF-STEM image, (b) Cu-*K* STEM-EDX map, and (c) Ru-*L* STEM-EDX map of the Cu/$Cu_{1-x}Ru_x$ NCs. (d) Reconstructed overlay image of the maps shown in panels (b) and (c) (blue, Cu; white, Ru).

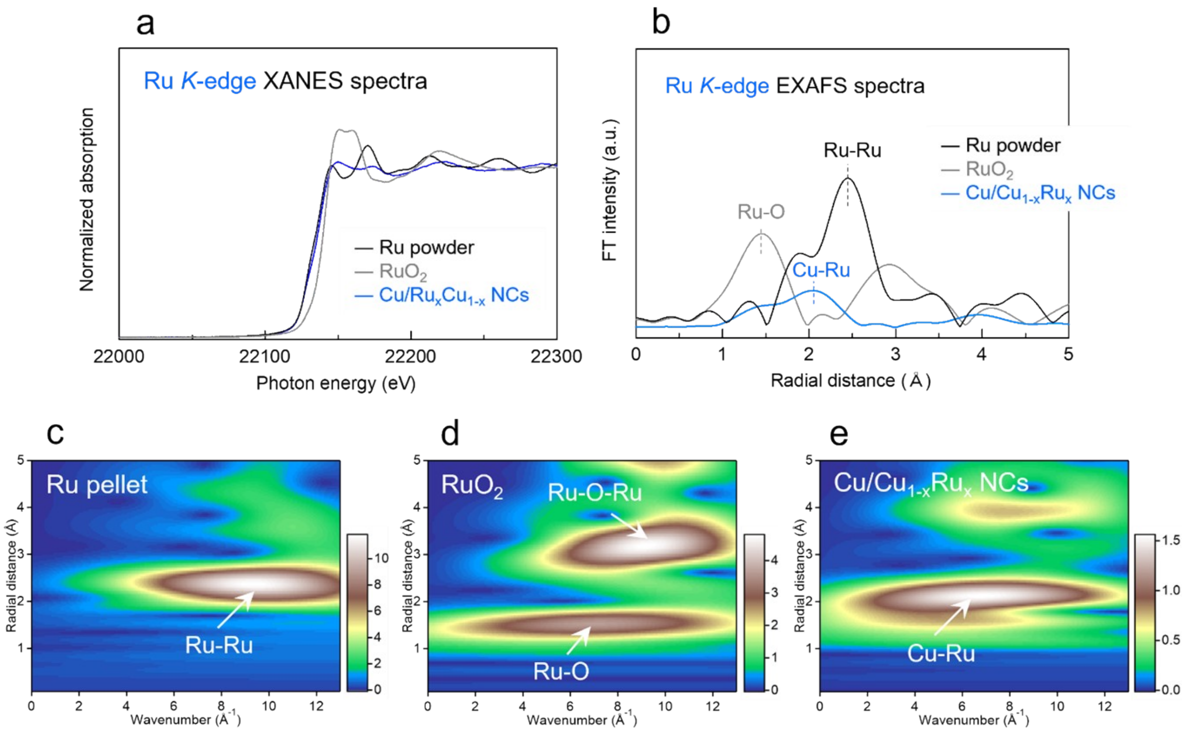

Figure S23. (a) XANES and (b) EXAFS spectra at the Ru *K*-edge of Cu/$Cu_{1-x}Ru_x$ NCs Ru *K*-edge wavelet transform EXAFS of Ru pellet (c), RuO_2 powder (d) and Cu/$Cu_{1-x}Ru_x$ NCs (e). The colour bar represents the intensity range. □

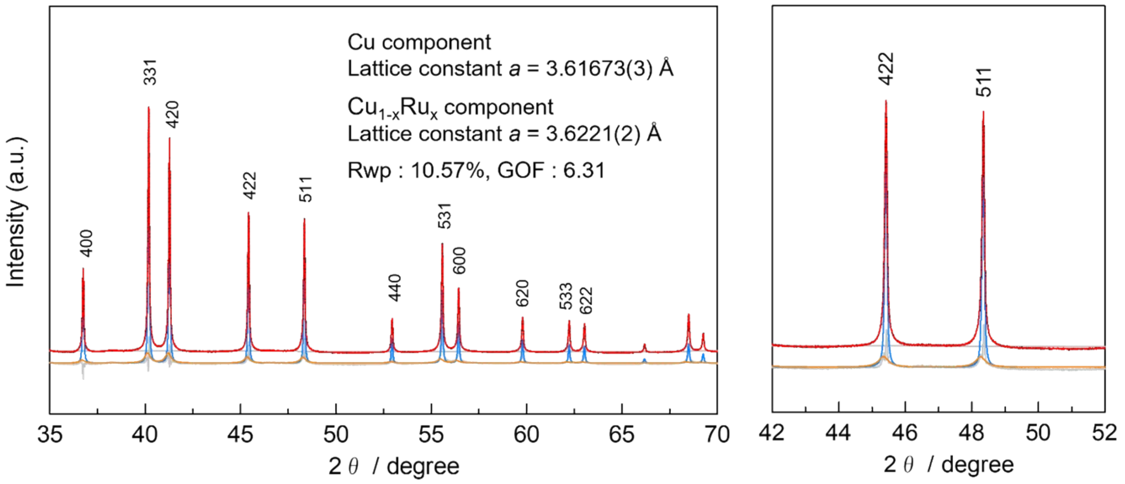

Figure S24. Synchrotron XRD pattern of Cu/$Cu_{1-x}Ru_x$ NCs (black line) at 303 K, and calculated pattern (red dots). The bottom lines show the difference profile (grey) and the fitting curves of the Cu (blue) and $Cu_{1-x}Pt_x$ (orange) components. The radiation wavelength was 0.5699 Å. Assuming that the lattice constant (3.6221(2) Å) follow Vegard's law, the minor component corresponds to $Cu_{0.95}Ru_{0.05}$ solid solution alloys.

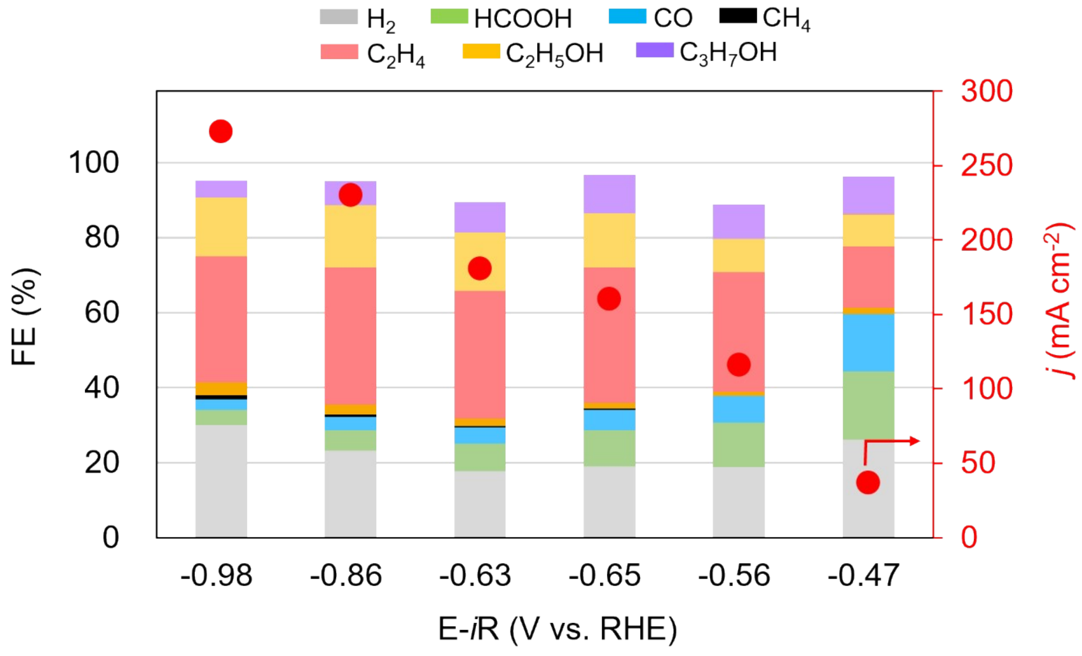

Figure S25. FEs of CO_2RR products on Cu/$Cu_{1-x}Pd_x$ NCs at various potentials.

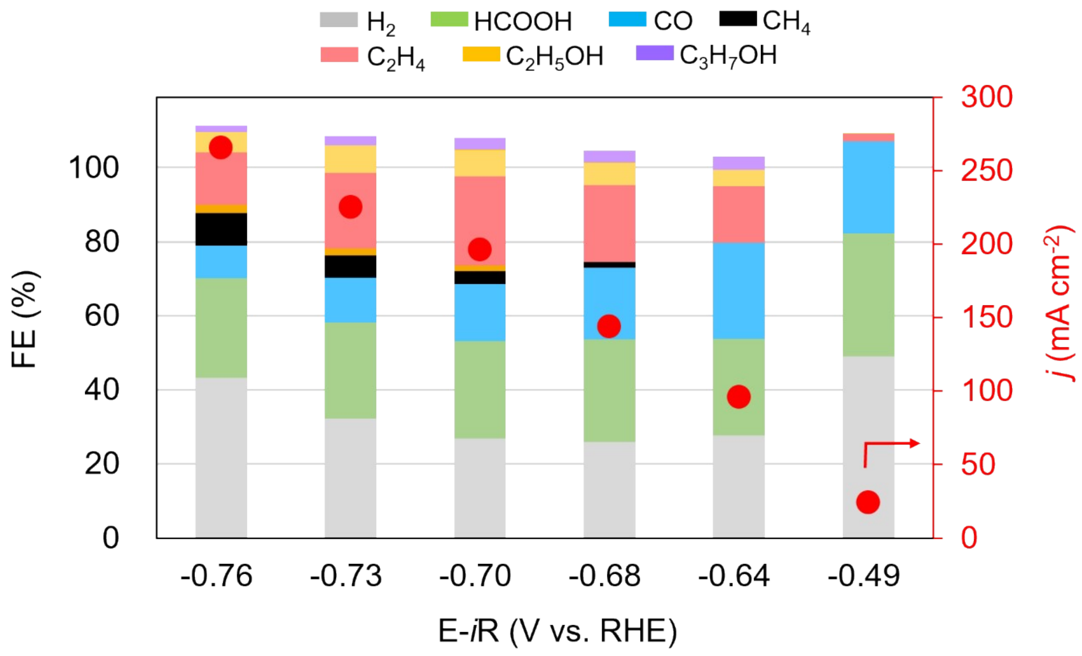

Figure S26. FEs of CO_2RR products on Cu/$Cu_{1-x}Pt_x$ NCs at various potentials.

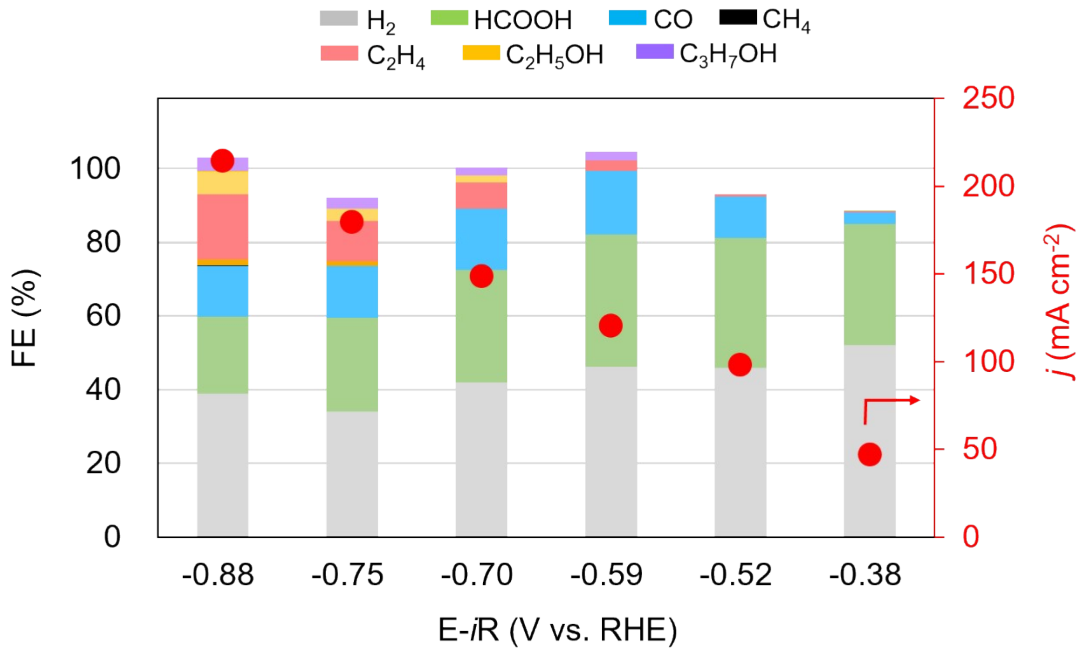

Figure S27. FEs of CO_2RR products on Cu/$Cu_{1-x}Ru_x$ NCs at various potentials.

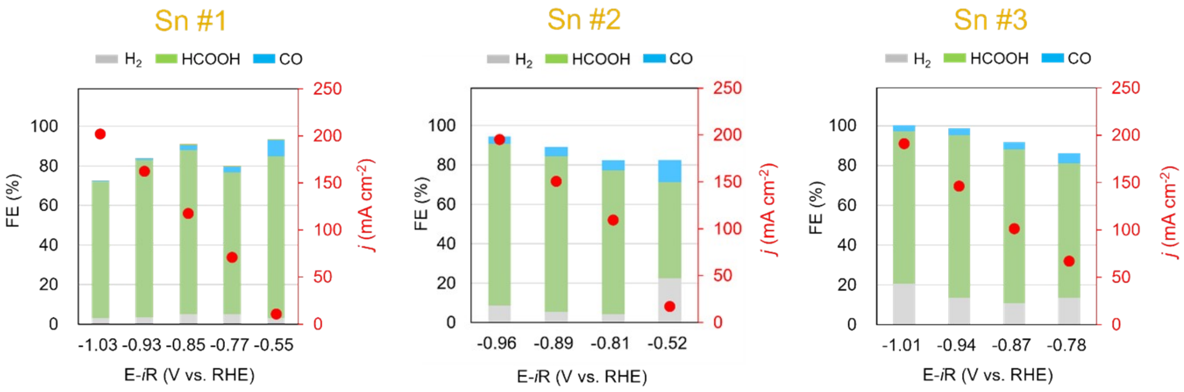

Figure S28. FEs of CO_2RR products on three Sn NPs at various potentials.

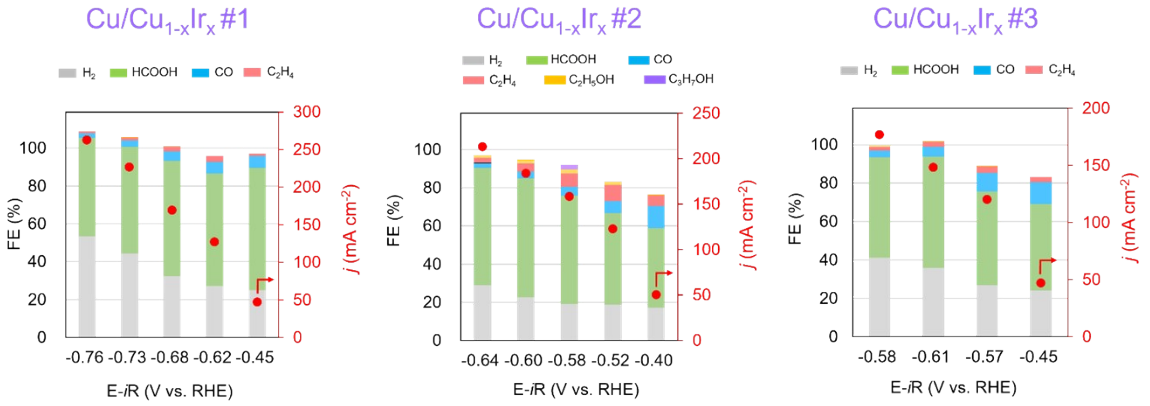

Figure S29. FEs of CO_2RR products on three $Cu/Cu_{1-x}Ir_x$ NCs at various potentials. The data of $Cu/Cu_{1-x}Ir_x$ #1 corresponds to Fig. 3b.

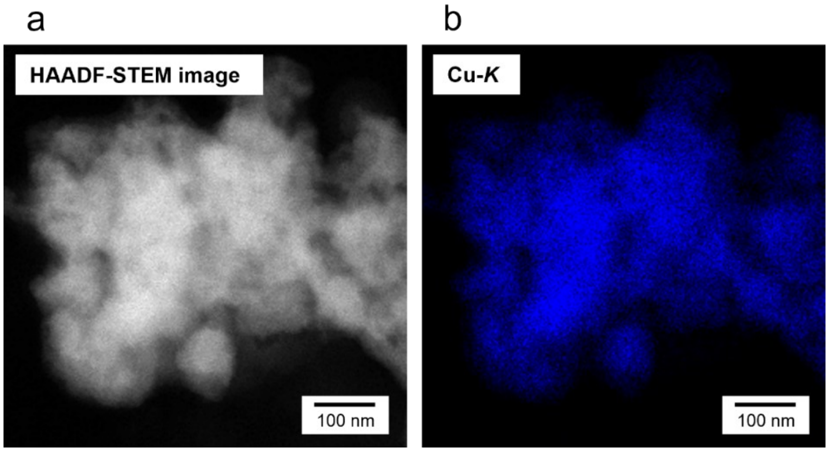

Figure S30. (a) HAADF-STEM image, (b) Cu-*K* STEM-EDX map of Cu NCs after CO_2RR.

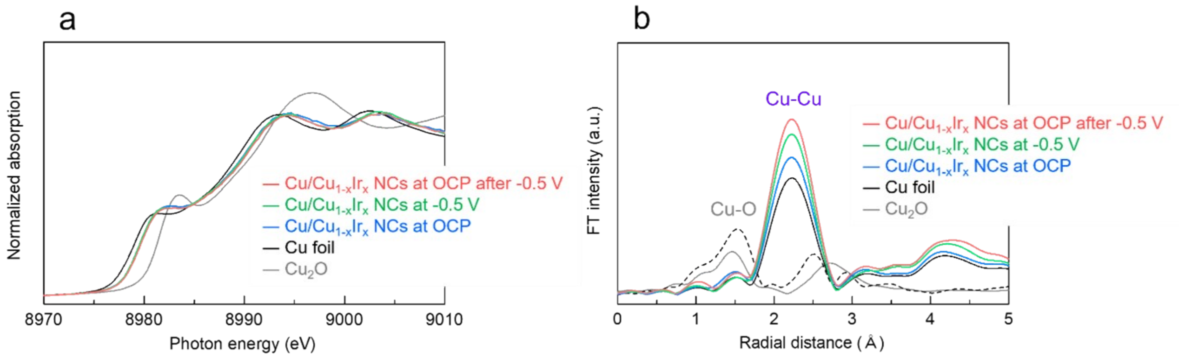

Figure S31. (a) in situ XANES and (b) EXAFS spectra at the Cu *K*-edge of $Cu/Cu_{1-x}Ir_x$ NCs.

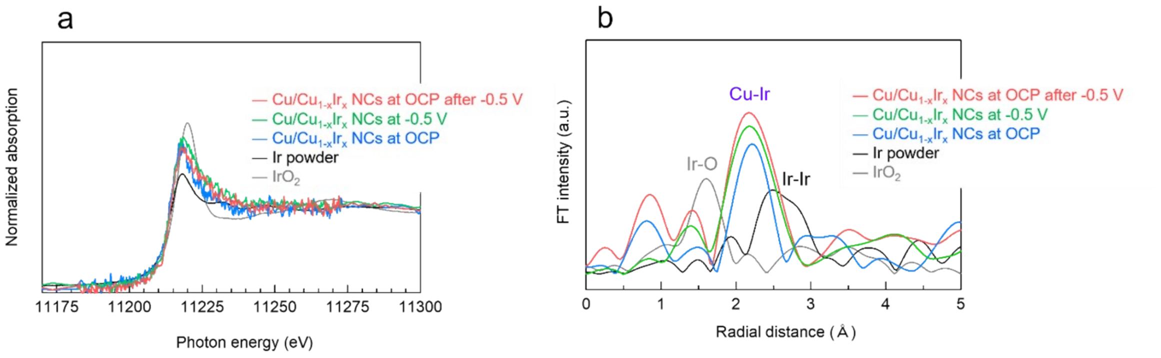

Figure S32. (a) in situ XANES and (b) EXAFS spectra at the Ir L_3-edge of Cu/$Cu_{1-x}Ir_x$ NCs.

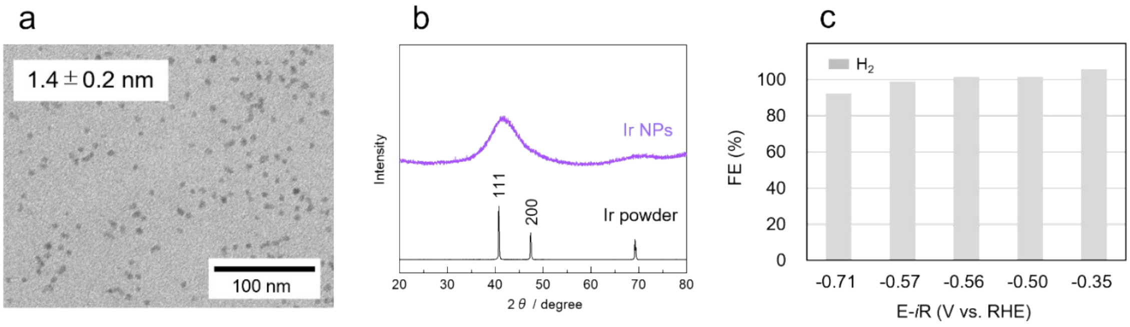

Figure S33. (a) TEM image, (b) XRD pattern and (c) FEs of CO_2RR products for Ir NPs. An aqueous solution of $IrCl_3$ (282 mg) in 210 ml of ethanol was refluxed for 3 hours with vigorous stirring. The solid was collected by evaporation.

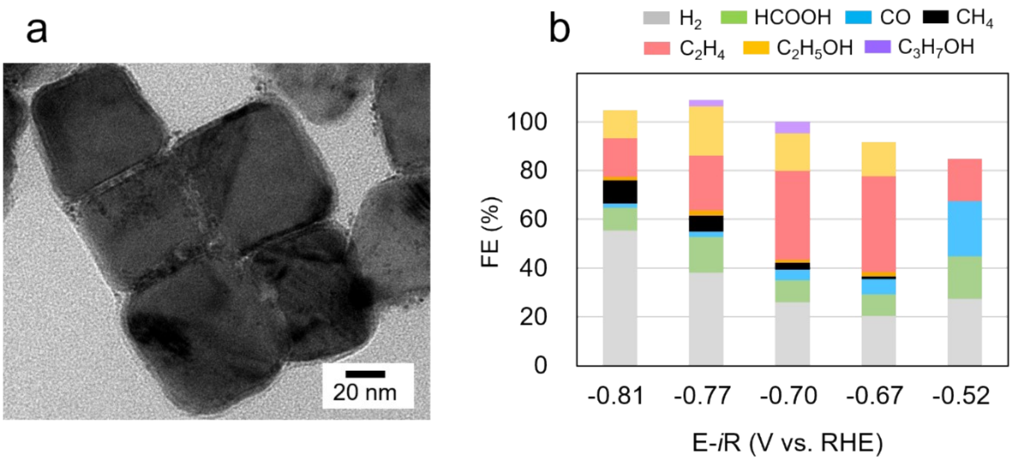

Figure S34. (a) TEM image (b) FEs of CO_2RR products for mixture of Cu NCs and Ir NPs. Cu NCs and Ir NPs were mixed in a 1 ml toluene solution and ultrasonically treated for 15 min. Ir content included in the mixture was estimated to be 0.7 at% from SEM-EDX.

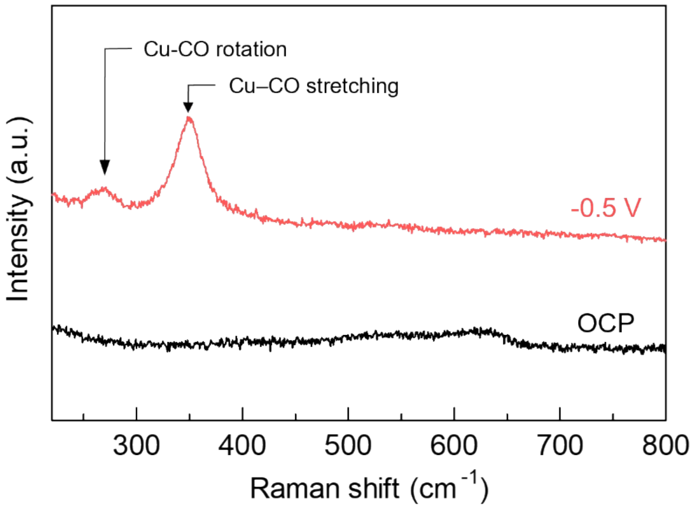

Figure S35. in situ Raman spectra recorded at OCV and -0.5 V vs. RHE for Cu/$Cu_{1-x}Pd_x$ NCs.

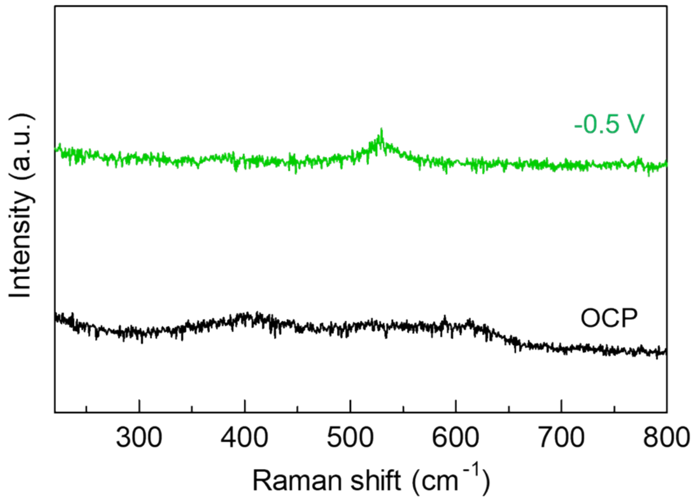

Figure S36. in situ Raman spectra recorded at OCV and -0.5 V vs. RHE for $Cu/Cu_{1-x}Pt_x$ NCs.

Table S5. Adsorption energy of CO_2 and H on Cu(100) and CuM(100) (M = Pd, Pt, and Ir) models by DFT calculations.

E_{ads} (eV)	Cu	CuPd	CuPt	CuIr
CO_2	-0.252	-0.304	-0.307	-0.373
H	-0.393	-0.447	-0.570	-0.845
$\Delta E_{ads}(CO_2 - H)$	0.141	0.143	0.263	0.472

Table S6. Adsorption energy of COO, COOH and HOOH on Cu(100) and CuM(100) (M = Pd, Pt, and Ir) models by DFT calculations.

E_{ads} (eV)	Cu	CuPd	CuPt	CuIr
COOH	-0.124	-0.093	-0.726	-1.312
HCOO	-0.719	-0.421	-0.674	-0.989
HCOOH	-0.754	-0.764	-0.837	-1.014

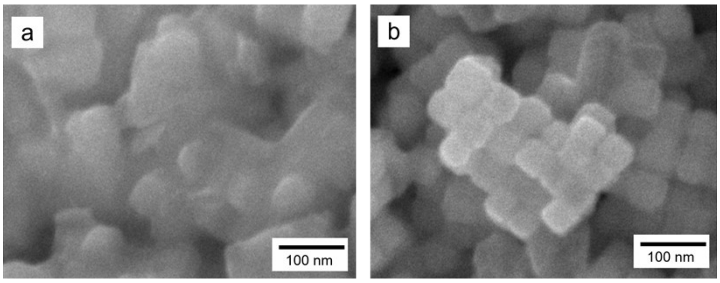

Figure 37. SEM images of (a) $Cu/Cu_{1-x}Pd_x$ and (b) $Cu/Cu_{1-x}Pt_x$ NCs after CO_2RR.

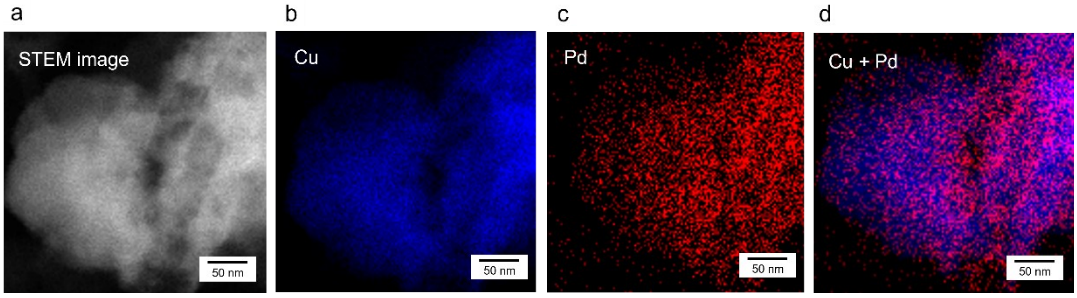

Figure 38. STEM image and EDX maps of $Cu/Cu_{1-x}Pd_x$ NCs after CO_2RR.

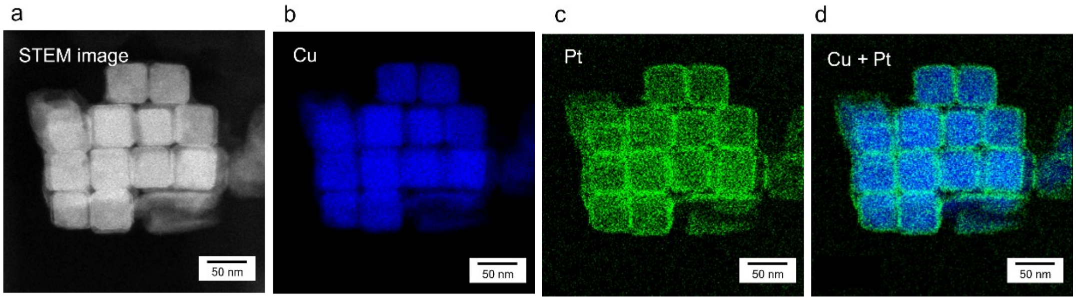

Figure 39. STEM image and EDX maps of $Cu/Cu_{1-x}Pt_x$ NCs after CO_2RR.

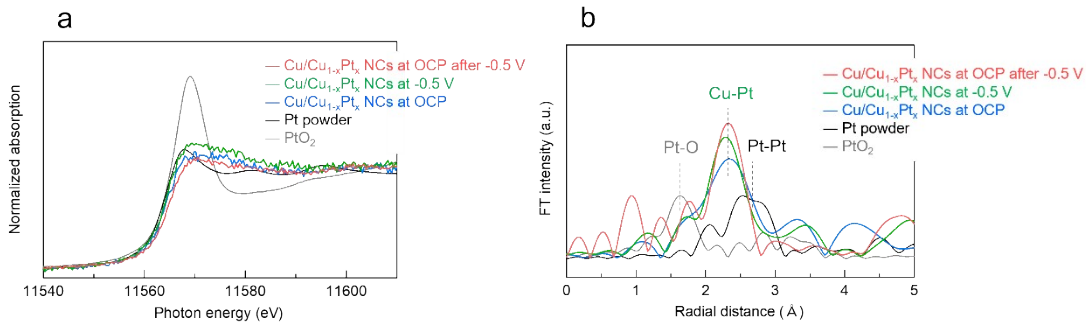

Figure 40. (a) in situ XANES and (b) EXAFS spectra at the Pt L_3-edge of $Cu/Cu_{1-x}Pt_x$ NCs

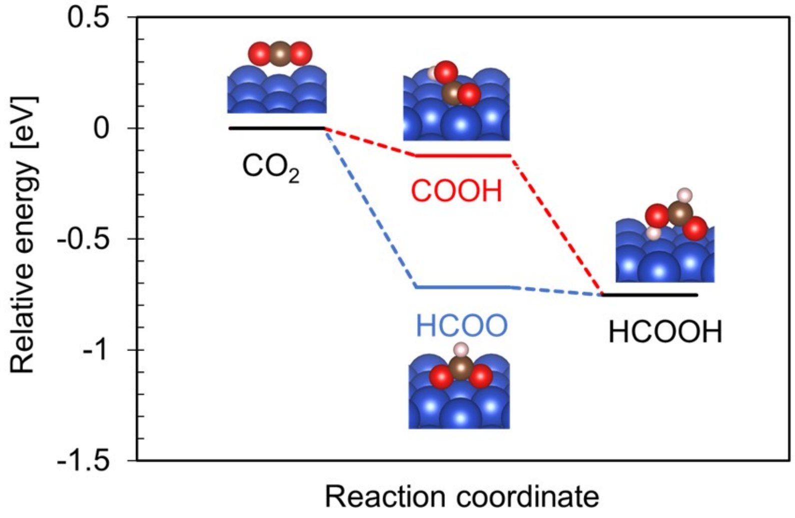

Figure S41. Energy profile of CO_2RR on Cu(100).

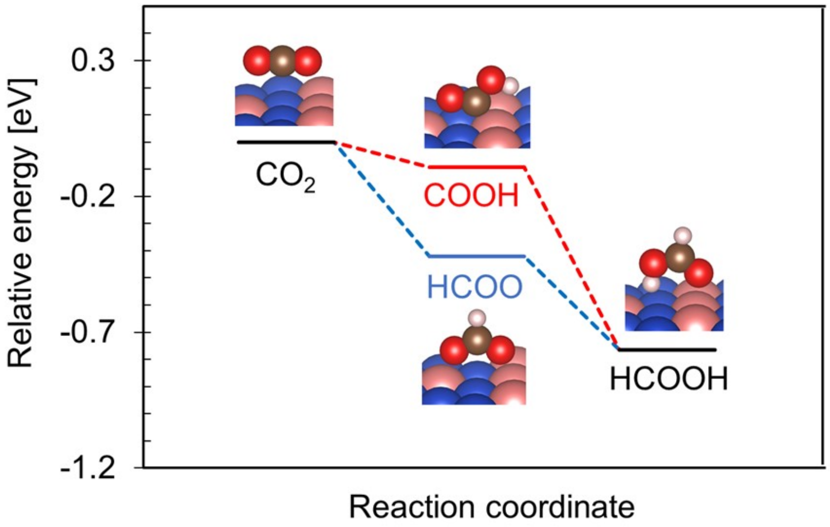

Figure S42. Energy profile of CO_2RR on CuPd(100).

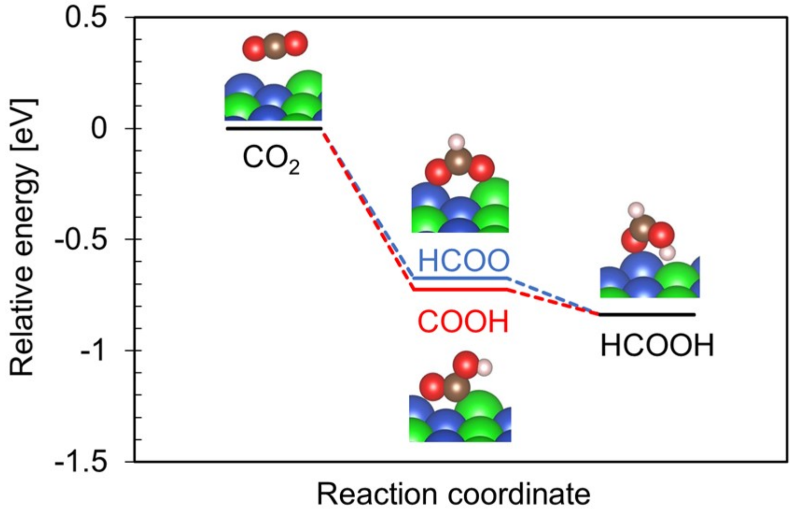

Figure S43. Energy profile of CO_2RR on CuPt(100).

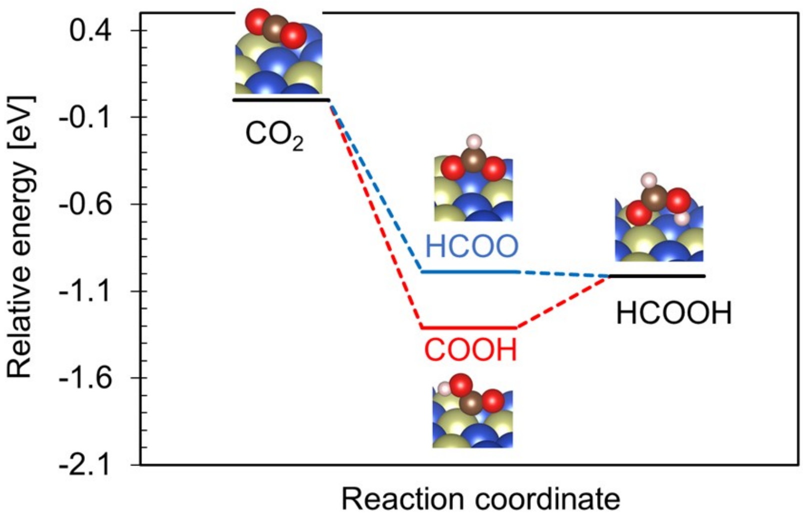

Figure S44. Energy profile of CO_2RR on CuIr(100).

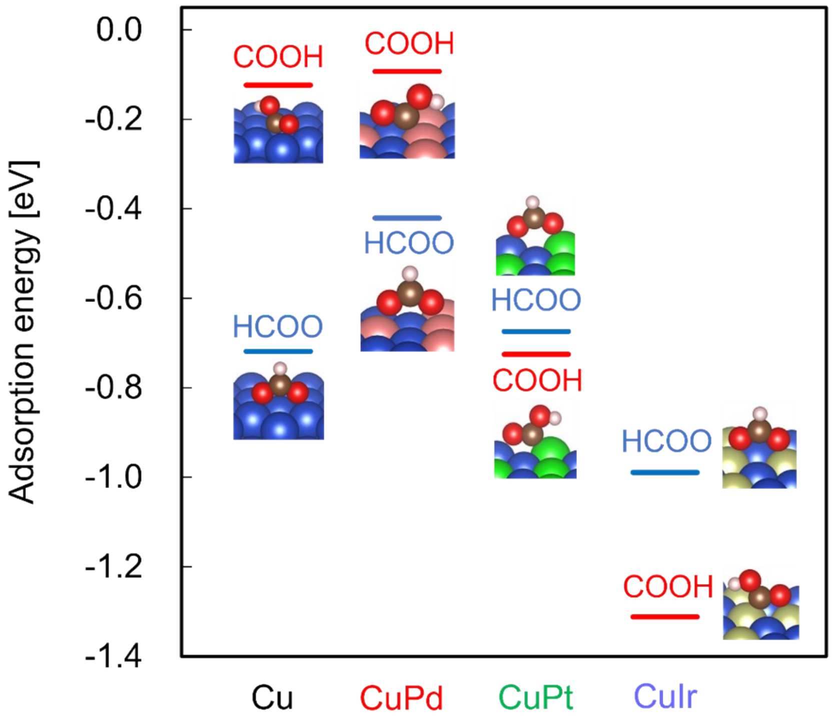

Figure S45. Stability of intermediates by DFT calculation.
